# Systematic characterization of position one variants within the lantibiotic nisin

**DOI:** 10.1038/s41598-018-37532-4

**Published:** 2019-01-30

**Authors:** Marcel Lagedroste, Jens Reiners, Sander H. J. Smits, Lutz Schmitt

**Affiliations:** Institute of Biochemistry, Heinrich-Heine-University Duesseldorf, Universitaetsstrasse 1, 40225 Duesseldorf, Germany

## Abstract

Lantibiotics are a growing class of natural compounds, which possess antimicrobial activity against a broad range of Gram-positive bacteria. Their high potency against human pathogenic strains such as MRSA and VRE makes them excellent candidates as substitutes for classic antibiotics in times of increasing multidrug resistance of bacterial strains. New lantibiotics are detected in genomes and can be heterologously expressed. The functionality of these novel lantibiotics requires a systematic purification and characterization to benchmark them against for example the well-known lantibiotic nisin. Here, we used a standardized workflow to characterize lantibiotics consisting of six individual steps. The expression and secretion of the lantibiotic was performed employing the promiscuous nisin modification machinery. We mutated the first amino acid of nisin into all proteinaceous amino acids and compared their bactericidal potency against sensitive strains as well as strains expressing nisin resistance proteins. Interestingly, we can highlight four distinct groups based on the residual activity of nisin against sensitive as well as resistant *L*. *lactis* strains.

## Introduction

Since the last decade the exponential increase of the number of antibiotic resistant strains steadily alarms the world health organization, which is reflected in their annually reports regarding the surveillance of antimicrobial and antibiotic resistance (WHO, GLASS report 2016–2017). Therefore, the urgent need for antimicrobial compounds, which can be used as alternatives to the classic antibiotic treatment, has dramatically increased. Some classes of antibiotic such as cephalosporins, macrolides, carbapenems or penicillin derivatives are vital for human medicine and the treatment of microbial infections. However, observed resistance to important antibiotic classes makes it necessary to explore new classes of natural or synthetic antimicrobial compounds^1^.

One possible class are antimicrobial peptides (AMP). Within this class especially lanthipeptides possessing antimicrobial activity, which are called lantibiotics (lanthionine containing antibiotics), are considered as possible lead compounds^2^. Lantibiotics are ribosomally synthesized as a precursor peptide (LanA), between 30–60 amino acids in size and undergo specific post-translational modifications (PTM)^3,4^. They are furthermore dissected into an N-terminal leader peptide and a C-terminal core peptide, in which the PTMs are installed by specialized modification enzymes. Upon leader peptide cleavage, the lantibiotic becomes activated and exhibits antimicrobial activity with efficiencies in the low nanomolar to millimolar range. Lantibiotics like NAI-107 or NVB302 are already subjected to pre-clinical trails and might be good candidates in the treatment of multidrug-resistant strains like MRSA or similar Gram-positive strains^5–7^.

Up to now >50 different lantibiotics with a similar set of PTMs have been described (for more details see review^8^). One specific PTM within the core peptide of lantibiotics is the dehydration of serine and threonine residues resulting in the formation of dehydroalanine and dehydrobutyrine residues. This reaction is catalysed by a specific dehydratase called LanB (dehydratase of class I lantibiotics)^9^. The hallmark of lantibiotics is the second modification, which leads to the formation of lanthionine (Lan) and/or methyl-lanthionine (MeLan) rings. The reaction proceeds via a Michael-type condensation of the dehydrated serine or threonine residues with the thiol group of a cysteine residue, which is introduced regio- and stereospecifically by the cyclase LanC (cylcase of class I lantibiotics)^10^. This ring formation results in a thioether bond giving rise to high thermostability and more profound resistance against proteolytic degradation. Furthermore, antimicrobial activity strictly depends on the presence of the Lan/MeLan rings^11^.

In general, lantibiotics exercise their antimicrobial activity through different modes of action. One prominent mode is the binding to the pyrophosphate moiety of lipid II molecules concomitant with the inhibition of peptidoglycan biosynthesis^12^. Another mode of action is the perturbation of bacterial cell membranes. Nisin or subtilin build a pore-forming complex with its receptor lipid II thereby sequestering lipid II within the membrane^13,14^ (for a review about lipid II binding peptides see^11^). In the case of nisin, which is active in the low nM range, this process occurs on the ms time scale^15,16^. Some lantibiotics such as for example Pep5 however, directly penetrate the target membrane^17^.

In comparison to other cationic AMPs, cytotoxicity against human cells is rarely observed, since lipid II, the main target of lantibiotics, is absent in eukaryotic membranes (except the two-peptide lantibiotic cytolysin S/L^18^). Thus, the efficient activity and the low cytotoxicity combined with few examples of known inherent resistances (see^19–21^) constitute these peptides as excellent lead structures for new antibiotics.

Nevertheless, the bottleneck of lantibiotic research is the identification and characterization of these compounds. With respect to the first, lantibiotics can be detected in genome sequences by data-mining approaches using bioinformatic tools such as BAGEL4^22^, antiSMASH^23^, RiPPquest^24^ and RiPPMiner^25^. Such tools either detect open reading frames encoding lantibiotics within a genome based on neighbouring PTM-enzymes or combine specific sequences of PTM-enzymes and the cleavage motifs within the leader peptides.

However, every newly identified lantibiotic requires a detailed experimental characterization with respect to its antimicrobial properties. Therefore, the isolation of a native sample or heterologously expressed samples in Gram-negative bacteria (*E*. *coli*)^26,27^ or Gram-positive bacteria (*L*. *lactis* or *B*. *subtilis*)^28–30^ is a prerequisite. Several reports indicate that the nisin modification and secretion system can be employed to modify and secrete other lantibiotics. Apparently, the nisin PTM system provides sufficient promiscuity to produce for example bagelicin from *Streptococcus suis* R61, flavucin from *Corynebacterium lipophiloflavum* and others peptides, if their core peptide is fused to the nisin leader peptide^29,31,32^. Nisin, produced by the Gram-positive *Lactococcus lactis* (*L*. *lactis*) bacterium and modified by the nisin PTM machinery is one, if not the best characterized lantibiotic and may therefore be used as a standardized lantibiotic for benchmarking.

Since the number of novel lanthipeptides/lantibiotics increases due to genome mining, design of hybrid-peptides (by coupling different lanthipeptides to a certain leader peptide), simple mutations or even chemical synthesis, a general pipeline to characterize the potential antimicrobial activity and thereby potency of a lantibiotic is urgently required to ensure appropriate benchmarking of such lantibiotics^1,33,34^.

**Figure 1:**
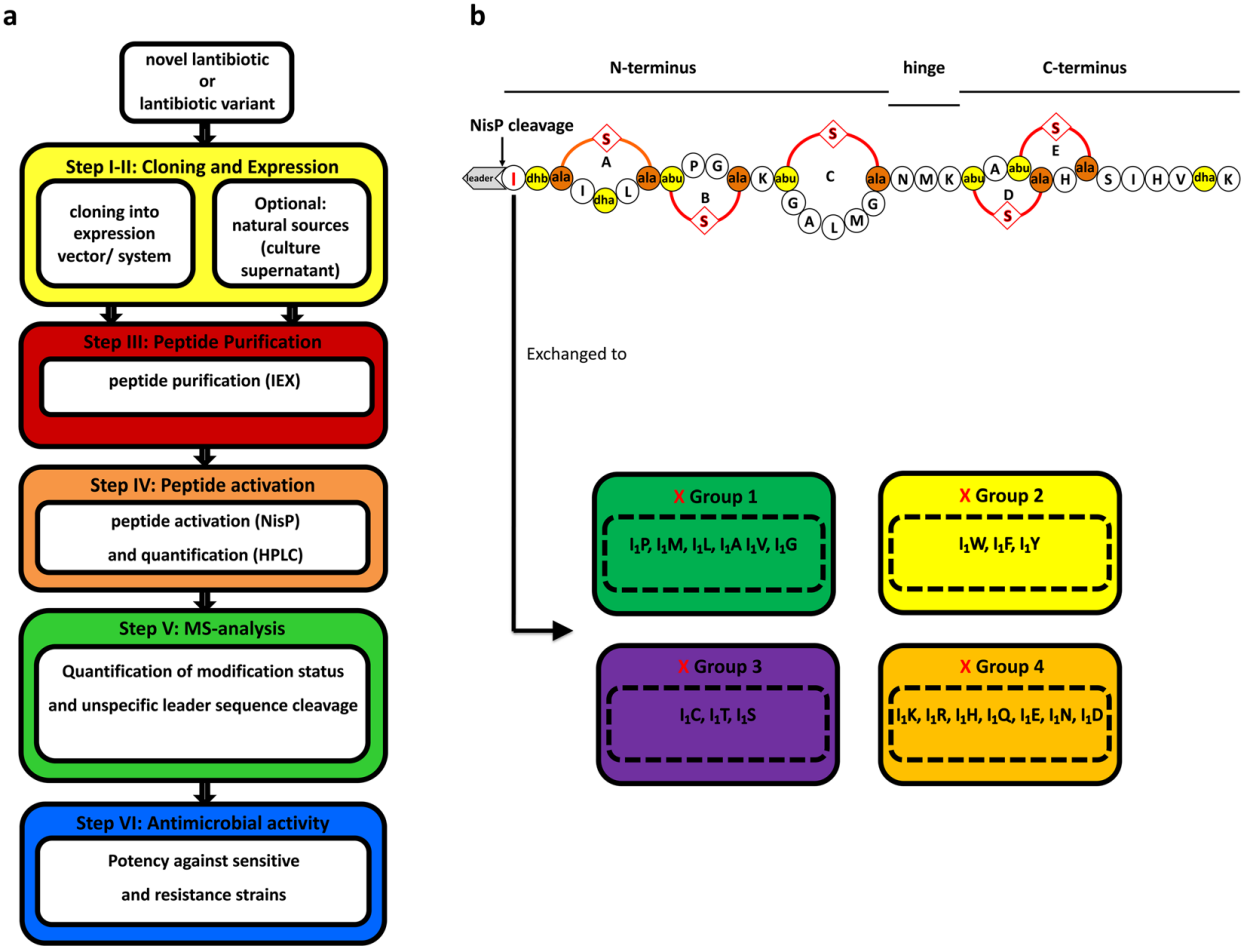
Scheme of lantibiotic characterization (**a**) the lantibiotic nisin and its I_1_ mutants (**b**). The characterization of novel lantibiotics or variants is based on a six steps protocol (**a**). I-II are cloning and expression steps (yellow box). Step III covers peptide purification (red box). Step IV represents the activation of the peptide (orange box). Step V is the MS-analysis (green box), while step VI represents the antimicrobial activity of the lantibiotic (blue box). The lantibiotic nisin (NisA) can be dissected into an N-terminal region (with lanthionine ring A and the methyl-lanthionine rings B and C), a hinge region and a C-terminal region (with the intertwined methyl-lanthionine rings D and E) (**b**). The dehydrated amino acids dehydroalanine (dha) and dehydrobutyrine (dhb) (former serines and threonines) are highlighted in yellow. The coupled cysteine residues to dehydrated amino acids are highlighted in orange. The thioether bonds between the (methyl-) lanthionine are marked with a red S. The position one isoleucine is highlighted in red and exchanged to X amino acid from the four different groups of natural amino acids.

Here, we used a standardized workflow for the characterization of lantibiotics, containing up to six steps depending on the availability of the lantibiotic (Fig. 1a). We exchanged the isoleucine at position one (I_1_) of nisin to all other 20 natural occurring amino acids (aa) (Fig. 1b) and determined the impact on the expression, modification and antimicrobial properties of these nisin variants and benchmarked it against wild type nisin.

## Results

### General characterisation of nisin I1 mutants

The nisin A core peptide was a target of many former studies aiming to alter the antimicrobial activity of nisin or to broaden its spectrum against microbial targets. Position 1 in the nisin core peptide received our attention since some mutations were described that lacked in our opinion a complete and quantitative characterization. For example, the substitutions of tryptophan-analogues^35^ as well as the mutants I_1_W, I_1_K, I_1_D^36^, I_1_G and I_1_Q (described in^37^). Accordingly, we characterized all mutants at I_1_ of nisin A using our standardized protocol, which is based on six individual steps (Fig. 1a; I-VI).

The first steps (I-III) concerning expression, secretion and purification of the nisin variants gave no major variation compared to wild type nisin, which were all expressed as a leader-containing variant (pre-nisin). The yield of pre-nisin was 6.0 ± 0.3 mg/ L culture supernatant (Supplementary Fig. S1) with a purity >95% based on Tricine-SDS-PAGE (Supplementary Fig. S2). The yield of most I_1_ mutants decreased to 40–60% of wild type nisin (Supplementary Fig. S1). The mutants I_1_C and I_1_W had an even lower yield of approximately 35%. This lower yield however did not affect our purification protocol and all pre-nisin variants were purified using the same protocol and resulted in comparable purity (Supplementary Fig. S2)^29,32^.

Step IV concerns leader peptide cleavage and thereby activation of the purified pre-nisin variants. Here, we used the secreted variant of the natural leader peptidase NisP, by which the leader peptide is cleaved *in vitro* and the cleavage efficiency (%) can be calculated as recently shown^38^. We used an RP-HPLC set-up to monitor product formation, in which the appearance of the leader peptide in the chromatograms was used to determine the concentration of activated nisin (Supplementary Fig. S3). Pre-nisin (Supplementary Fig. S3) had a retention time (RT) between 19 to 21 min, whereas the leader peptide eluted as two peaks corresponding to the variants with and without the N-terminal methionine between 14 to 16 min, respectively. The nisin core peptide eluted at a later retention time of approximately 23 min (Supplementary Fig. S3). In some cases, the nisin I_1_ mutants resulted in separated peaks of pre-peptide and core peptide after the activation by NisP (compare mutants I_1_M, I_1_L, I_1_V, I_1_W, I_1_Y, I_1_F and I_1_C in Supplementary Fig. S4). However, in the case of the I_1_ mutants I_1_A, I_1_G, I_1_T, I_1_S, I_1_K, I_1_R, I_1_H, I_1_Q, I_1_E, I_1_N and I_1_D) the formation of a core peptide peak in terms of a new and separated peak in the chromatogram was not observed (Supplementary Fig. S4). Rather, the peaks with a retention time between 18–21 min contained both species, the cleaved and non-cleaved lantibiotic, and can therefore not be deconvoluted for proper determination of the amount of activated nisin. Hence, only the two peaks of the leader peptide were used and the area was integrated for the determination of the concentration of activated nisin and its I_1_ mutants. The concentration was calculated based on a standard calibration curve (Supplementary Fig. S5), where we precisely determined the concentration of the activated nisin or I_1_ mutants. We used this concentration determination instead of the colorimetric protein concentration determination as for example Bradford or the BCA-assay since the latter two assays cannot differentiate between the activated and non-activated form of the secreted variants. Since nisin is not active unless the leader is cleaved off, the determination of the area of the leader peak to calculate the amount of the activated lantibiotic directly relates to the antimicrobial activity without need to further purify the peptide after cleavage.

All I_1_ mutations were subjected to NisP cleavage and most of the variants were cleaved, but a strong influence of the nature of the I_1_ substitutions with respect to cleavage efficiency was observed (Supplementary Fig. S6). The cleavage efficiency for wild type pre-nisin A was 94.3 ± 1.7% but lower for the mutants I_1_M, I_1_L, I_1_A and I_1_V (72.3 ± 1.4%, 72.4 ± 1.3%, 81.0 ± 1.8% and 58.1± 2.8%, respectively). All other variants displayed efficiency below 50% (Supplementary Fig. S6). This holds especially in those cases, where the amino acid at position one was exchanged to a charged amino acid (e.g. I_1_K 9.2 ± 1.3%; I_1_E 9.5 ± 0.7%) or contained a bulky hydrophobic side chain (e.g. I_1_F 12.9 ± 0.8%; I_1_W 5.7 ± 0.2%; I_1_Y 22.6 ± 2.3%) (Supplementary Fig. S6). To highlight two examples, NisP was only able to cleave 5.7 ± 0.2% of the I_1_W variant, which was significantly lower as the cleavage efficiency previously reported^35,39^. Interestingly, the I_1_P mutant was not cleaved at all by NisP (Supplementary Figs S4 and S6). Even after an extensive prolongation of the incubation time, no leader peptide peak was detected in the RP-HPLC chromatogram (Supplementary Fig. S4). It is important to stress in this context that the cleavage efficiencies cannot be determined from Tricine-SDS gels (20%). Here, the leader peptide as well as the core peptide would co-migrate, which obviously will falsify the staining results.

As previously reported^37,38^, a prerequisite for high cleavage efficiency by NisP is the presence of at least one (methyl-)lanthionine ring. Therefore, we wondered whether some of the nisin variants with low cleavage efficiencies were altered in their modification status (step V). To detect possible ring formation(s) we incubated the lantibiotic prior to MS analysis with the thiol-reactive agent 1-cyano-4-dimethylaminopyridinium tetrafluoroborate (CDAP), which binds to free cysteines and results in a mass shift of 25 Da per covalently attached CDAP. When no mass shift occurs, all cysteine residues are part of thioether rings, while for every mass shift of 25 Da one cysteine is not part of a (Me)Lan thioether ring. As controls, we also used the unmodified version of pre-nisin (Supplementary Fig. S7a), in which no rings are present and five CDAP adducts were identified indicating that five cysteine side chains were available for a simultaneous labelling reaction and the fully modified pre-nisin, showing no coupling products (Supplementary Fig. S7b). This highlights the quantitative nature of the CDAP assay^31^.

For most analysed I_1_ mutants no CDAP attachment was observed (Supplementary Figs S8, S9, S10 and Supplementary Table S1). Exceptions of this observation are the mutants I_1_L, I_1_A, I_1_V, I_1_G, I_1_F, I_1_Y, I_1_R, I_1_H, I_1_Q, I_1_N, where small amounts of coupling products were observed with variations from 7x dehydrations (dh) to 5x dh with one coupling product (Supplementary Figs S8, S9, S10 and Table S1). These amounts are very small, in comparison to the main species and the MS analysis showed clearly, that the I_1_ mutants primary containing all lanthionine rings. In the case of nisin I_1_C, where one additional cysteine residue was introduced also one coupling product was observed (Supplementary Fig. S9) In summary, although the analysis of the cleavage reaction revealed I_1_ mutants with lower cleavage efficiency, the modifications of the core peptide within these variants were not altered.

### Impact of mutations at position I_1_ on antimicrobial activity of nisin

Growth inhibition assays (step VI) were used to determine the potency of the activated I_1_ mutants. First, this assay was performed against the sensitive strain NZ9000-Cm harbouring an empty plasmid pIL-SV (the strain NZ9000-Erm gave identical results) to determine the value, at which 50% of the cells were inhibited in growth (IC_50_ value).

**Figure 2:**
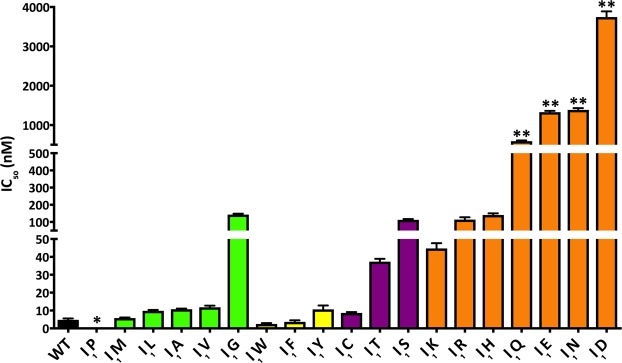
Growth inhibition assay of strain NZ9000-Cm in the presence of nisin and the corresponding I_1_ variants. The lantibiotic nisin A (WT) and its I_1_ mutants were used for growth inhibition (IC_50_) against strain NZ9000-Cm. IC_50_ values were grouped in four different sub-groups (group 1: aa M-G, green; group 2: aa W-Y, yellow; group 3: aa C-S, magenta; group 4: aa K-D, orange), Values represents the average of at least five independent measurements and the errors report the standard deviation of the mean (SDM). The nisin variant I_1_P was not cleaved by NisP (*) and no growth inhibition assay was conducted. The nisin variants within the forth group marked with (**) showed less antimicrobial activity (IC_50_ > 500 nM).

The nisin A wild type (WT) had an IC_50_ value of 4.8 ± 0.7 nM, which is in a similar range as previous reported values determined with strains NZ9000-Cm/NZ9000-Erm^40–42^ (Fig. 2 and Supplementary Table S2). The I_1_P mutant was used in very high concentrations (>1 mM) in the growth inhibition assay but displayed no antimicrobial activity (Fig. 2; IC_50_ value is marked with a star symbol). This indicated that this variant was not activated by NisP, in line with our observations described above.

All other variants were used and displayed IC_50_ values ranging from wild type level to 100 nM or even to lower µM values. Based on the measured activities, the variants can be grouped into four classes depending on the amino acid property.

The first group contains mainly aliphatic amino acids (except M)(Fig. 2, green bars and Supplementary Table S2). The mutation to methionine gave similar IC_50_ values to WT (5.8 ± 0.3 nM), but the exchange to the amino acids leucine, alanine and valine lead to a two-fold decrease in activity (9.8 ± 0.5 nM, 10.7 ± 0.4 nM and 11.8 ± 0.9 nM, respectively). The mutation to glycine lead to an even further decrease in activity and the IC_50_ value was determined to 143.0 ± 5.1 nM.

The second group (Fig. 2, yellow bars and Supplementary Table S2) contained aromatic amino acids (except histidine, which belongs to the fourth group) and displayed a high antimicrobial activity against the sensitive strain. Especially, the mutants I_1_W and I_1_F displayed lower IC_50_ values (2.5 ± 0.2 nM and 3.7 ± 0.8 nM) than WT, indicating an increased antimicrobial activity. The mutant I_1_Y showed a lower activity and displayed an IC_50_ value of 10.6 ± 0.9 nM.

The third group (Fig. 2, magenta bars and Supplementary Table S2) displayed IC_50_ values ranging from 8.6 to 112 nM. The mutation I_1_C lead to antimicrobial activity of WT (below 10 nM) with an IC_50_ value of 8.6 ± 0.5 nM, whereas the mutation to I_1_T or I_1_S displayed a considerable reduction of the antimicrobial activity with IC_50_ values of 37.3 ± 1.6 nM and 112.4 ± 5.0 nM, respectively. Interestingly, these substitutions are all amino acids, which are potential targets of the PTM machinery. Since the mutation I_1_C introduced an additional cysteine residue, we carefully analysed for the presence of an additional (Me)Lan ring. However, the coupling assay clearly demonstrated the presence of one accessible cysteine suggesting that this nisin variant still harbours five (Me)Lan rings (Supplementary Fig. S9). Unfortunately, this assay cannot determine which ring(s) are formed. Since the IC_50_ value is not altered, we propose that this mutant follows normal ring formation and the additional introduced cysteine at position one was labelled with CDAP.

The mutations I_1_T and I_1_S, containing an additional dehydration position. Based on our MS analysis, we saw mainly no additional dehydration, which leads to the conclusion that this position is not well recognized by NisB (Supplementary Fig. S9). But we clearly observed that the possible higher number of dehydrations of the peptide at the N-terminus of the core peptide decreased antimicrobial activity (IC_50_ values > 30 nM). Here, mutation to the polar residue serine had a larger impact on the antimicrobial activity of the core peptide compared to the threonine substitution.

The fourth group (Fig. 2, orange bars and Supplementary Table S2) contained charged amino acids and the amide side chains of glutamate and aspartate. In general, a strong negative effect on the antimicrobial activity was observed within this group. The IC_50_ values were higher compared to the other groups and ranged from 44 nM to 3746 nM. The substitutions at position 1 to the amino acids lysine, arginine or histidine lead to 10-fold or even 20-fold higher IC_50_ values (I_1_K: 44.7 ± 3.0 nM, I_1_R: 113.9 ± 13.6 nM and I_1_H: 140.0 ± 5.0 nM) compared to the WT IC_50_ value. A major alteration in antimicrobial activity was observed in the case of an exchange I_1_Q and I_1_E, respectively. Here, the IC_50_ values were 592.0 ± 17.8 nM for I_1_Q and 1328.0 ± 32.7 nM for I_1_E. An even more dramatic effect was observed upon introduction of I_1_N or I_1_D. Here, IC_50_ values of 1386.0 ± 46.3 nM and 3746.0 ± 144.1 nM were determined. These variants displayed such high IC_50_ values (marked by two star symbols (**)) that these mutations were not analysed in further growth inhibition assays using resistant strains.

In summary, the antimicrobial activity of the different I_1_ mutants towards the sensitive strain NZ9000-Cm correlated with the physico-chemical properties of amino acid at position one. Although this substitution was only one amino acid and the modification state of the core peptide was not altered, the active lantibiotics showed drastic variations in antimicrobial activities. Based on the growth inhibition assay, these I_1_ mutants were divided into four groups, where aromatic amino acids at position one lead to higher activity but an introduction of polar, charged amino acids or its amidated counterparts decreased the potency of the corresponding variant to the sensitive strain.

### The influence of I_1_ mutants on immunity and resistance proteins

Lantibiotics are regarded as potential antibiotic candidates, which might have the potential to replace classic antibiotics and thereby overcome the increasing resistances against major antibiotic classes. One potential drawback of lantibiotics is highlighted by the few reported resistance mechanisms against for example nisin^43^. Therefore, it is critical, to screen for strains, which might be resistant against the new lantibiotic, if a new lantibiotic is characterized and its potency is determined. In our protocol, we implemented first the screen against a sensitive strain (see above), but more importantly we included four strains expressing immunity or resistance proteins against nisin. The immunity proteins NisI (lipoprotein) and NisFEG (ABC transporter) from L. *lactis* are the first and second line of defence of the nisin producer strain^44^. Upon expression in the sensitive strain NZ9000, these proteins might provide immunity and one can study the activity of the lantibiotic in the presence of immunity proteins. Additionally, we screened the effect of the I_1_ variants on the nisin resistance proteins *Sa*NSR (lipoprotein) and *Sa*NsrFP (ABC transporter) to fully consider the potency of the newly lantibiotic/lantibiotic variant. If these proteins are expressed in the sensitive strain, they confer resistance against the nisin and likely to nisin variants.

**Figure 3:**
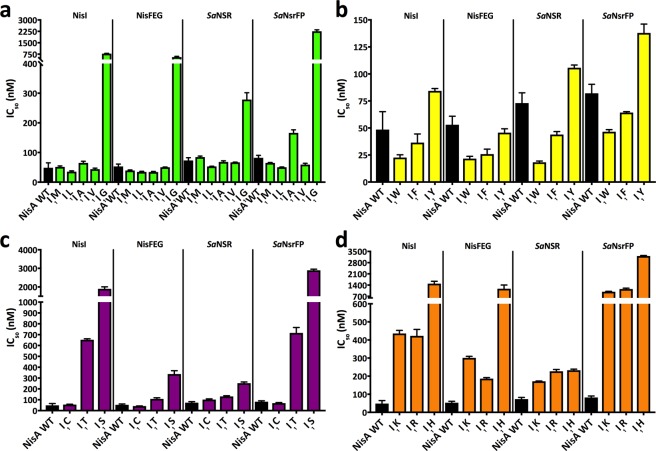
Growth inhibition assay of strains NZ9000-NisI, NZ9000-NisFEG, NZ9000-*Sa*NSR and NZ9000-*Sa*NsrFP in the presence of nisin and the corresponding I_1_ variants. The lantibiotic nisin A (WT) and its I_1_ mutants were used for growth inhibition (IC_50_) with strains NZ9000-NisI, NZ9000-NisFEG, NZ9000-*Sa*NSR and NZ9000-*Sa*NsrFP. The IC_50_ values were grouped in sub-groups of the I_1_ mutants. The group 1 with the aa M-G (**a**), group 2 with the aa W-Y (**b**), group 3 with the aa C-S (**c**) and group 4 with the aa K-H (**d**). Values represent the average of at least five independent measurements and the errors report the standard deviation of the mean (SDM).

The nisin variants were analysed according to the above provided classification of four groups. The first group of I_1_ mutants showed a similar tendency in activity towards the strains for immunity and resistance as for the sensitive strain (Supplementary Table S2). The substitutions of isoleucine to methionine, leucine, valine and alanine gave IC_50_ values for strain NZ9000-NisI in a range of 35–65 nM and were comparable to nisin A WT (46.0 ± 6.0 nM) (Fig. 3a). Only the substitution to glycine gave a higher value of 785.7 ± 9.7 nM reflecting the low activity of this variant. The IC_50_ values of the first group for the strain NZ9000-NisFEG were also in the range of 34–50 nM (nisin A WT: 53.0 ± 4.5 nM). Again, a higher IC_50_ value of 557.8 ± 28.3 nM was determined for the I_1_G mutant.

When analysing the results of the resistance strains two major changes were observed. In general, the IC_50_ values of the I_1_ mutants for strain NZ9000-*Sa*NSR were similar (53–84 nM) to the WT (73.1 ± 3.6 nM). The IC_50_ value for the I_1_G mutant was 278.5 ± 13.3 nM and lower in comparison to the IC_50_ values of the other strains (Fig. 3a). The IC_50_ values for strain NZ9000-*Sa*NsrFP changed only for the mutants I_1_A (166.1 ± 5.1 nM) and I_1_G (2257.0 ± 53.4 nM), respectively, reflecting the lower activity of these variants towards the strain.

The second group of I_1_ mutants included the aromatic amino acids, for which a higher activity against the immunity and resistance strains was observed (Supplementary Table S2). The higher activity was determined for all strains (I_1_W > I_1_F > wild type > I_1_Y). The exception is the mutant I_1_Y and strain NZ9000-NisFEG. Here, the IC_50_ value was lower than the one of nisin A WT (53.0 ± 4.5 nM) (Fig. 3b). Interestingly, the mutant I_1_W showed high potency for the strains NZ9000-NisI, NZ9000-NisFEG and NZ9000-*Sa*NSR with IC_50_ values below 25 nM. A higher IC_50_ value of 46.6 ± 1.1 nM was only determined against strain NZ9000-*Sa*NsrFP (Fig. 3b).

The third group of I_1_ mutants showed opposing results concerning immunity and resistance (Supplementary Table S2). Here, mutants I_1_T and I_1_S had lower antimicrobial activity than wild type reflected by IC_50_ value even above 100 nM (Fig. 3c). Especially, the more than five-fold lower activity against strains NZ9000-NisI and NZ9000-*Sa*NsrFP is surprising as the IC_50_ values were determined to be above 500 nM. For the mutant I_1_T the values are 653.3 ± 5.1 nM and 716.1 ± 28.6 nM, whereas for the mutant I_1_S the values are two-fold higher (1898.0 ± 62.3 nM and 2893.0 ± 34.8 nM) (Fig. 3c). The exception is the mutant I_1_C, which showed a similar activity towards the strains as nisin A WT with IC_50_ value in the range of 41 to 101 nM. Based on the MS analysis only one free cysteine was present, we cannot conclude which of the cysteines is not part of a thioether bridge. As this variant showed a similar antimicrobial activity, we however suggest no alteration in ring pattern (Supplementary Fig. S9).

In the last group of I_1_ mutants, all variants showed lower antimicrobial activity towards the strains of immunity and resistance (Supplementary Table S2). The IC_50_ values were above 400 nM (Fig. 3d) except for mutants I_1_R and I_1_K, which had IC_50_ values of 186.4 ± 3.1 and 301.2 ± 4.7 nM, respectively, against strain NZ9000-NisFEG. Similar to this, mutants I_1_R, I_1_K and I_1_H showed a higher antimicrobial activity against strain NZ9000-*Sa*NSR (Fig. 3d).

**Figure 4:**
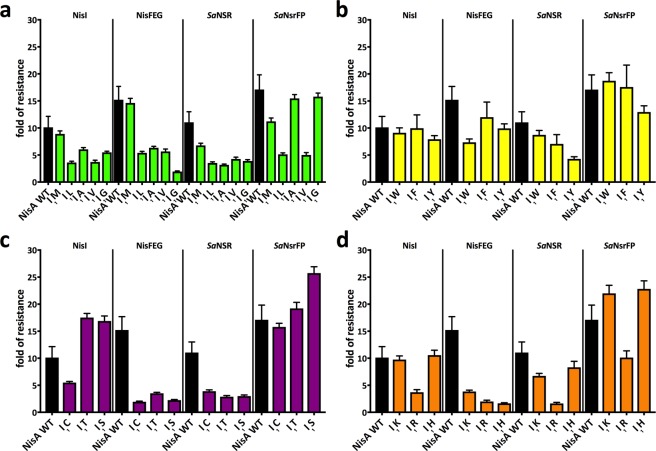
Fold of resistance of strains NZ9000-NisI, NZ9000-NisFEG, NZ9000-*Sa*NSR and NZ9000-*Sa*NsrFP in comparison to NZ9000-Cm. The resistance of the strains NZ9000-NisI, NZ9000-NisFEG, NZ9000-*Sa*NSR and NZ9000-*Sa*NsrFP towards the lantibiotic nisin A (WT) and its I_1_ mutants are presented as fold of resistance. The fold of resistance values were grouped in sub-groups of the I_1_ mutants. The group 1 with the aa M-G (**a**), group 2 with the aa W-Y (**b**), group 3 with the aa C-S (**c**) and group 4 with the aa K-H (**d**). Values represent the average of at least five independent measurements and the errors report the standard deviation of the mean (SDM).

In summary, I_1_ mutations clearly influenced the efficiency of the immunity and resistance proteins. The IC_50_ values of the sensitive strain and resistance strains for the different I_1_ mutations can be used to calculate a fold of resistance (Fig. 4). Here, the effect of certain mutations is even less pronounced (e.g. I_1_S or I_1_T on NZ9000-NisI) but still show the reduced activity. The reasons for this reduction might be that these variants are better ligand and/or substrates for the immunity and resistance proteins. Another explanation might be, that the variants have an altered interaction with the membrane (I_1_K; I_1_R, I_1_W, I_1_F). The same is true for the opposite case, in which the mutants are more active. Here, the mutants are either worse substrates or showed a loss in membrane attraction due to charge repulsion (I_1_E/D mutants) or reduced hydrophobicity (I_1_G mutant).

## Discussion

The increased detection of antibiotic resistances of human pathogenic strains urgently calls for the identification of novel lead structures, which can be used to develop long lasting antibiotics. One promising family of candidates are the antimicrobial peptide subfamily of lantibiotics. They are small ribosomally synthesized and post-translational modified peptides^45,46^, which possess a potent antimicrobial activity generally in the nM range. Their antimicrobial activity makes them excellent candidates to treat MDR-strains such as MRSA or VRE^47^. Recently, numerous new lantibiotics were discovered by “*in silico* genome mining” approaches, using available bacterial genome sequence data^48^. Here, BAGEL4 and its predecessor BAGEL3 are powerful algorisms to detect lantibiotics sequences within bacterial genome sequences^22^. Novel lantibiotic such as flavucin, bagelicin and agalacticin were found and their antimicrobial properties were determined^29^. In the study by Heel *et al*. 2016 the potency of many new lantibiotics were screen against a set of Gram-positive and Gram-negative strains to show their antimicrobial potencies. Till now, however, it is not possible to deduce the potency solely on sequence information and every lantibiotic needs to be expressed and purified for subsequent characterization of its antimicrobial activity. To ensure comparability of the determined activities and the potencies derived from these experiments a standardized protocol needs to be established, which allows benchmarking novel against already characterized lantibiotics (e.g. nisin). This should also include mutations designed or natural variants of previously characterized lantibiotics.

In this study, we used a standardized protocol based on six individual steps (I-VI). Some of these steps have been previously (partly) published, but not as a combined robust protocol^29,32,40,42,49^. By replacing I_1_ of nisin A against any other amino acid, the complete influence of the exchange on expression, purification and/or activity was monitored to provide a quantitative characterisation.

The characterisation of a novel lantibiotic starts with the expression system and the choice of homologous or heterologous expression. Homologous expression of a lantibiotic is often associated with its isolation from supernatants of lantibiotic producer strains (examples are epidermin^50^, mutacin 1140^51^, NAI-107 and related lantibiotics^52^ or pinensins^53^). The yield of this strategy can be limited, especially if the producer is hardly cultivable under lab conditions or induction of the lantibiotic is not trigged (e.g. geobacillin I^54^, salivaricin 9^55^ and staphylococcin Au-26,^56^).

Therefore, for some lantibiotics such as lichenicidin from *Bacillus licheniformis* ATCCC 14580^26^, or prochlorosins from *Prochlorococcus* MIT 9313^57^ a heterologous expression in *E*. *coli* is the preferential strategy to obtain higher yields of the pre-lantibiotic. Alternatively, the expression via the NICE-system in *L*. *lactis*^58^ is a possibility. Here, the pre-lantibiotic is modified after induction with the lantibiotic nisin by the PTM system (NisB/NisC) and secreted by the ABC transporter NisT^32^.

The nisin PTM system was shown to be of sufficient promiscuity to modify and secrete lantibiotics as well as non-lantibiotic peptides^29,32^. As an advantage any lantibiotic can be produced as a pre-lantibiotic. This results in higher yields, as the lantibiotic is not antimicrobial active and is not limiting the growth of the producer strain. Furthermore, the pre-lantibiotic (as pre-nisin) shows higher pH stability in contrast to nisin (low solubility at pH > 7)^59^. Normally, we observed yields for the pre-nisin and its variants of 3–6 mg per liter cell culture supernatant after purification (Supplementary Fig. S1). Considering these yields further down-streaming steps are employed to characterize the lantibiotic.

Although NisB and NisC are promiscuous in modifying their substrate, it is crucial that both enzymes fully modify the core peptide similar to pre-nisin. Otherwise, the steps III (cleavage by NisP) and VI (antimicrobial assay) might be less informative. Thus, even new mutants of nisin, like mutants in the hinge region^60,61^ or in the leader peptide^37^, required a full characterisation with respect to their modification and antimicrobial activity.

The purified pre-lantibiotics need to be analysed by RP-HPLC before and after activation by proteolytic cleavage of the leader peptide. In this study, we activated pre-nisin and the I_1_ mutants *in vitro* by purified leader peptidase NisP (Supplementary Figs S3 and S4; described in^38,49^). The cleavage efficiency and also the exact concentration of the active lantibiotic were determined via RP-HPLC (Supplementary Fig. S6). Hence, every activation of the pre-lantibiotic can be monitored and quantified by integrating the leader peptide peaks, which are used for calculation of the amount of activated lantibiotic. We observed for some I_1_ mutants, that the properties during the RP-HPLC runs of the pre-lantibiotics and activated lantibiotics made it impossible to distinguish between both peaks (Supplementary Fig. S4). Consequently, the areas of leader peptide peaks were used for quantification. One other aspect that has to be taken in consideration is the fact that for nisin mutants, such as I_1_A or I_1_K, the core peptide cannot be purified by means of preparative RP-HPLC from the supernatant. In general, the analytic RP-HPLC is needed to quantify the lantibiotic and as a quality control for the purification and the cleavage reaction.

The major advantage of RP-HPLC compared to other technics is the more accurate determination of the concentration of the activated lantibiotic, which is a prerequisite for a reliable determination of the biological activity of the lantibiotic. In comparison to methods determining the total amount of peptide/protein (e.g. BCA-assay and other colorimetric assays) the determination via HPLC minimizes the error. For example, if only 15% of a pre-lantibiotic is cleaved, but the total amount of pre-lantibiotic is used for the calculations, the determined IC_50_ value would be six to seven-fold larger, which obviously indicates a falsely lower antimicrobial activity.

Sometimes a lower cleavage efficiency of the leader peptidase and later a lower antimicrobial activity might reflect incomplete modification of the peptide (e.g. dehydrations or the lack of lanthionine rings) or disrupted recognition by the leader peptidase. As an example, the mutation of I_1_ in nisin Z to tryptophan leads to the production of two variants. The I_1_W mutant as a main product and a small amount of I1W/Dh2T, where the threonine residue escape the dehydration and an altered activity is proposed^62^. Therefore, a proper determination of dehydration and the formation of lanthionine rings by MS-analysis is required. MS-analysis provides information about the dehydration reaction of NisB^63^ by the loss of water (−18 Da), but cannot indicate the formation of the (methyl-)lanthionine rings. Furthermore, it is not possible to distinguish between the position of the dehydration within the nisin core peptide, which might have escaped modification like threonine position 2 or serine position 33^64^. Thus, it is inevitable to use at least one orthogonal method to determine the presence of lanthionine ring(s). Either the use of an alkylation agent (e.g. CDAP to couple the free thiol group from cysteine residues) or tandem MS-MS analysis to determine the alternate fragmentation pattern between WT pre-lantibiotic and potential mutant variants is necessary.

In our protocol we choose the CDAP-coupling assay to detect nisin I_1_ mutants, which might still possess free thiol groups. The advantage of the CDAP-coupling assay is the additional mass shift in the MS spectra. Here, a mass shift of 25 Da per thiol-conjugated easily indicates incomplete ring-formation.

The activated lantibiotic was used for antimicrobial activity assays (step VI), in which different indicator strains were employed. Here, the assay for antimicrobial activity needs to be suitable for the characterisation of the lantibiotic. The broth dilution with 2-fold dilution series of lantibiotics in a MIC assay is generally used to determine the antimicrobial activity^65^. However, if only one stock concentration is used, one obvious limitation is the factor two in serial dilutions, which will detect only changes larger than two. Therefore, we suggest to use the IC_50_ assay with a high diversity of stock concentrations to screen every data point. Still, both methods have their limit as no information about the mode of action of the lantibiotic can be acquired.

The active lantibiotic were screened against a sensitive strain to determine the IC_50_ value by growth inhibition assay (described with modifications in^41^). We used the *L*. *lactis* strain NZ9000 with the corresponding empty vector systems pNZ-SV or pIL-SV, termed NZ9000-Erm and NZ9000-Cm, respectively. Generally, we would like to propose that the strain *L*. *lactis* NZ9000 is used as a standard strain, in order to have comparable values from antimicrobial activity assay.

The full potential of a lantibiotic or lantibiotic variants can be shrouded if the screen is only performed against sensitive strains. Therefore, based on the IC_50_ value from the sensitive strains, a second screen against strains, which express immunity or resistance proteins should be performed^66^. We used the strains NZ9000-NisI and NZ9000-NisFEG to study the effect of the immunity proteins from *L*. *lactis* as they confer immunity via different mechanisms^67,68^. To analyse the effect of a lantibiotic on resistance potency, strains NZ9000-*Sa*NSR and NZ9000-*Sa*NsrFP expressing proteins involved the nisin resistance from *Streptococcus agalactiae* were used^42,66^. Importantly, the ratio of the IC_50_ values (fold of resistance) of these strains to the sensitive strain describes the alteration of substrate specificity on the immunity or resistance proteins (described in^40^).

As final remark, the production of lantibiotics, especially if they are heterologously expressed, has to be benchmarked against a standardized lantibiotic. Here, the well-studied lantibiotic nisin can be used again to benchmark against novel and unknown lantibiotics.

In summary, our analysis and the suggested roadmap demonstrate that a detailed and multifaceted investigation of the antimicrobial potency of lantibiotic is necessary to uncover the full potential of novel lantibiotics, but also to quantitatively compare the efficiency of different members of this promising family of antimicrobial peptides.

## Materials and Methods

### Microorganisms and culture conditions

Strains and the plasmids used in this study are listed in Table S3. Cultures of *L*. *lactis* NZ9000 were grown in M17 medium at 30 °C supplemented with 0.5% glucose (GM17 and the appropriate antibiotics (final concentrations 5 µg/ml). In the case of pre-lantibiotic secretion, the *L*. *lactis* strain NZ9000 was grown in minimal medium at 30 °C supplemented with 0.5% glucose and the appropriate antibiotics (final concentrations 5 µg/ml).

The cultures of the *E*. *coli* strain DH5α were grown in Luria-Bertani (LB) medium at 37 °C under aerobic conditions with the appropriate antibiotic (100 µg/ml ampicillin or 30 µg/ml kanamycin final concentration).

### Cloning of nisin variants

The substitution of amino acids in the *nisA* gene was performed by standard site-directed mutagenesis. Here, the vector pNZ-SV-nisA was used as a template to introduce mutations by Pfu-DNA polymerase (Thermo-Scientific) following standard protocol of the manufacture. Used oligonucleotides are listed elsewhere (Supplementary Table S4). Sequence analysis verified the correctness of the nisin variants and plasmids were transformed into electro-competent *L*. *lactis* NZ9000 strain, already containing the pIL3-BTC vector for expression.

### Expression and purification of NisP

The soluble variant of NisP was expressed by *L*. *lactis* NZ9000 harbouring the plasmid pNGnisP8His and subsequently purified as previously described^38^.

### Expression purification and activation of pre-nisin variants

Pre-nisin and its variants were expressed and purified as previously described^41,49,69^. The purity of pre-nisin and its variants was controlled by Tricine-SDS-PAGE (20%)^70^. The activation of all variants was performed by NisP cleavage overnight at 8 °C. Pre-nisin cleavage and its variants was monitored by RP-HPLC^49,69^.

### Determination of active nisin by HPLC analysis

Pre-nisin and the activated nisin variants were analysed by RP-HPLC (Agilent Technologies 1260 Infinity II) with a LiChrospher WP 300 RP-18 end-capped column and an acetonitrile/water solvent system. The cleavage efficiency and the concentration of active lantibiotic was calculated as described previously^49,69^.

### Cloning and expression of immunity and resistance proteins

The cloning of plasmids encoding for immunity and resistance proteins used in this study were previously described^40–42,71^. A nomenclature of the strains used for antimicrobial activity assay is shown elsewhere (Supplementary Table S3).

### Determination antimicrobial activity by growth inhibition assay

The growth inhibition assay with the NZ9000 strains was conducted as described in^41^. There, the inhibitory concentration (IC_50_), where only 50% of the cells survive can be calculated for the different lantibiotics.

The fold of immunity/resistance was calculated by dividing the IC_50_ values of strains expressing the immunity or resistance proteins from the IC_50_ value of the control strain harbouring the empty plasmid^40,42^.

### MALD-TOF analysis: Dehydration and lanthionine ring analysis

Prior to MS analysis the activated nisin variant were desalted via C_18_ ZipTip purification according to the manufactory manual (Merk-Millipore). The vacuum dried pellet was directly used for MALDI-TOF analysis or coupled with an organic coupling agent for free cysteine residues^72^. For coupling, the pellet was dissolved in 9 µl citrate buffer (25 mM, pH 3) and incubated with 2 µl TCEP (Tris[2- carboxyethyl]phosphine) (100 nmol) for 20 min at 29 °C. After the incubation, the coupling agent CDAP (1-cyano-4 dimethylaminopyridinium tetrafluoroborate) (140 nmol) was added and incubated for 15 min at 29 °C.

For MALDI-TOF analysis the vacuum dried pellet was dissolved with 50 µL 50% acetonitrile solution containing 0.1% TFA. From the soluble sample, 1 µl was mixed with 10 µl matrix solution (10 mg/ml alpha-cyano-4-hydroxycinnamic acid, dissolved in 50% acetonitrile containing 0.1% (v/v) trifluoroacetic acid) and 1 µl of the mixture was spotted on the target. The sample was analysed with MALDI-TOF (UltrafleXtreme, Bruker Daltonics, Bremen, Software: Compass 1.4) in positive mode.

## Supplementary information

Dataset 1

## References

[1] Tracanna V, de Jong A, Medema MH, Kuipers OP (2017). Mining prokaryotes for antimicrobial compounds: from diversity to function. FEMS Microbiol Rev.

[2] Dischinger J, Basi Chipalu S, Bierbaum G (2014). Lantibiotics: promising candidates for future applications in health care. Int J Med Microbiol.

[3] Schnell N (1988). Prepeptide sequence of epidermin, a ribosomally synthesized antibiotic with four sulphide-rings. Nature.

[4] Arnison PG (2013). Ribosomally synthesized and post-translationally modified peptide natural products: overview and recommendations for a universal nomenclature. Nat. Prod. Rep..

[5] Brunati C (2018). Expanding the potential of NAI-107 for treating serious ESKAPE pathogens: synergistic combinations against Gram-negatives and bactericidal activity against non-dividing cells. J Antimicrob Chemoth.

[6] Jabes D (2011). Efficacy of the New Lantibiotic NAI-107 in Experimental Infections Induced by Multidrug-Resistant Gram-Positive Pathogens. Antimicrobial Agents and Chemotherapy.

[7] Crowther GS (2013). Evaluation of NVB302 versus vancomycin activity in an *in vitro* human gut model of Clostridium difficile infection. J Antimicrob Chemoth.

[8] Repka LM, Chekan JR, Nair SK, van der Donk WA (2017). Mechanistic Understanding of Lanthipeptide Biosynthetic Enzymes. Chem Rev.

[9] Koponen O (2002). NisB is required for the dehydration and NisC for the lanthionine formation in the post-translational modification of nisin. Microbiology.

[10] Li B, van der Donk WA (2007). Identification of essential catalytic residues of the cyclase NisC involved in the biosynthesis of nisin. J Biol Chem.

[11] Oppedijk SF, Martin NI, Breukink E (2016). Hit ‘em where it hurts: The growing and structurally diverse family of peptides that target lipid-II. Biochim Biophys Acta.

[12] Hsu ST (2004). The nisin-lipid II complex reveals a pyrophosphate cage that provides a blueprint for novel antibiotics. Nat Struct Mol Biol.

[13] Hart P, Oppedijk SF, Breukink E, Martin NI (2016). New Insights into Nisin’s Antibacterial Mechanism Revealed by Binding Studies with Synthetic Lipid II Analogues. Biochemistry.

[14] Hasper HE, de Kruijff B, Breukink E (2004). Assembly and stability of nisin-lipid II pores. Biochemistry.

[15] Wiedemann I, Benz R, Sahl HG (2004). Lipid II-mediated pore formation by the peptide antibiotic nisin: a black lipid membrane study. J Bacteriol.

[16] Breukink E (1999). Use of the Cell Wall Precursor Lipid II by a Pore-Forming Peptide Antibiotic. Science.

[17] Brötz H (1998). Role of lipid-bound peptidoglycan precursors in the formation of pores by nisin, epidermin and other lantibiotics. Molecular Microbiology.

[18] Cox CR, Coburn PS, Gilmore MS (2005). Enterococcal cytolysin: A novel two component peptide system that serves as a bacterial defense against eukaryotic and prokaryotic cells. Curr Protein Pept Sc.

[19] Draper LA, Cotter PD, Hill C, Ross RP (2015). Lantibiotic resistance. Microbiol Mol Biol Rev.

[20] Khosa S, Hoeppner A, Gohlke H, Schmitt L, Smits SH (2016). Structure of the Response Regulator NsrR from Streptococcus agalactiae, Which Is Involved in Lantibiotic Resistance. PLoS One.

[21] Revilla-Guarinos A, Gebhard S, Mascher T, Zuniga M (2014). Defence against antimicrobial peptides: different strategies in Firmicutes. Environ Microbiol.

[22] van Heel, A. J. *et al*. BAGEL4: a user-friendly web server to thoroughly mine RiPPs and bacteriocins. *Nucleic Acids Res*, 10.1093/nar/gky383 (2018). 10.1093/nar/gky383 PMC6030817 29788290

[23] Blin K, Medema MH, Kottmann R, Lee SY, Weber T (2017). The antiSMASH database, a comprehensive database of microbial secondary metabolite biosynthetic gene clusters. Nucleic Acids Res.

[24] Mohimani H (2014). Automated Genome Mining of Ribosomal Peptide Natural Products. ACS Chemical Biology.

[25] Agrawal, P., Khater, S., Gupta, M., Sain, N. & Mohanty, D. RiPPMiner: a bioinformatics resource for deciphering chemical structures of RiPPs based on prediction of cleavage and cross-links. *Nucleic Acids Res*, 10.1093/nar/gkx408 (2017). 10.1093/nar/gkx408 PMC5570163 28499008

[26] Caetano T, Krawczyk JM, Mosker E, Sussmuth RD, Mendo S (2011). Heterologous expression, biosynthesis, and mutagenesis of type II lantibiotics from Bacillus licheniformis in Escherichia coli. Chem Biol.

[27] Tang W, van der Donk WA (2012). Structural characterization of four prochlorosins: a novel class of lantipeptides produced by planktonic marine cyanobacteria. Biochemistry.

[28] Sherwood EJ, Hesketh AR, Bibb MJ (2013). Cloning and analysis of the planosporicin lantibiotic biosynthetic gene cluster of Planomonospora alba. J Bacteriol.

[29] van Heel AJ (2016). Discovery, Production and Modification of Five Novel Lantibiotics Using the Promiscuous Nisin Modification Machinery. ACS Synth Biol.

[30] Chakicherla A, Hansen JN (1995). Role of the leader and structural regions of prelantibiotic peptides as assessed by expressing nisin-subtilin chimeras in Bacillus subtilis 168, and characterization of their physical, chemical, and antimicrobial properties. J Biol Chem.

[31] Kluskens LD (2005). Post-translational modification of therapeutic peptides by NisB, the dehydratase of the lantibiotic nisin. Biochemistry.

[32] Rink R (2005). Lantibiotic structures as guidelines for the design of peptides that can be modified by lantibiotic enzymes. Biochemistry.

[33] Ongey EL, Neubauer P (2016). Lanthipeptides: chemical synthesis versus *in vivo* biosynthesis as tools for pharmaceutical production. Microb Cell Fact.

[34] Burkhart BJ, Kakkar N, Hudson GA, van der Donk WA, Mitchell DA (2017). Chimeric Leader Peptides for the Generation of Non-Natural Hybrid RiPP Products. ACS Cent Sci.

[35] Zhou L (2016). Incorporation of tryptophan analogues into the lantibiotic nisin. Amino Acids.

[36] Montalban-Lopez M, Deng J, van Heel AJ, Kuipers OP (2018). Specificity and Application of the Lantibiotic Protease NisP. Front Microbiol.

[37] Plat A, Kluskens LD, Kuipers A, Rink R, Moll GN (2011). Requirements of the engineered leader peptide of nisin for inducing modification, export, and cleavage. Appl Environ Microbiol.

[38] Lagedroste, M., Smits, S. H. J. & Schmitt, L. Substrate Specificity of the Secreted Nisin Leader Peptidase NisP. *Biochemistry*, 10.1021/acs.biochem.7b00524 (2017). 10.1021/acs.biochem.7b00524 28675292

[39] Kuipers OP, Rollema HS, Beerthuyzen MM, Siezen RJ, de Vos WM (1995). Protein engineering and biosynthesis of nisin and regulation of transcription of the structural nisA gene. International Dairy Journal.

[40] AlKhatib Z (2014). The C-terminus of nisin is important for the ABC transporter NisFEG to confer immunity in Lactococcus lactis. Microbiologyopen.

[41] AlKhatib Z (2014). Lantibiotic immunity: inhibition of nisin mediated pore formation by NisI. PLoS One.

[42] Reiners J (2017). The N-terminal Region of Nisin Is Important for the BceAB-Type ABC Transporter NsrFP from Streptococcus agalactiae COH1. Front Microbiol.

[43] Khosa S, AlKhatib Z, Smits SH (2013). NSR from Streptococcus agalactiae confers resistance against nisin and is encoded by a conserved nsr operon. Biol Chem.

[44] Alkhatib Z, Abts A, Mavaro A, Schmitt L, Smits SH (2012). Lantibiotics: how do producers become self-protected?. J Biotechnol.

[45] Alvarez-Sieiro P, Montalban-Lopez M, Mu DD, Kuipers OP (2016). Bacteriocins of lactic acid bacteria: extending the family. Appl Microbiol Biot.

[46] Cotter PD, Hill C, Ross RP (2005). Bacteriocins: developing innate immunity for food. Nat Rev Microbiol.

[47] Piper C, Cotter PD, Ross RP, Hill C (2009). Discovery of medically significant lantibiotics. Curr Drug Discov Technol.

[48] van Heel AJ, de Jong A, Montalban-Lopez M, Kok J, Kuipers OP (2013). BAGEL3: automated identification of genes encoding bacteriocins and (non-)bactericidal posttranslationally modified peptides. Nucleic Acids Res.

[49] Abts A, Montalban-Lopez M, Kuipers OP, Smits SH, Schmitt L (2013). NisC binds the FxLx motif of the nisin leader peptide. Biochemistry.

[50] Bonelli RR, Schneider T, Sahl HG, Wiedemann I (2006). Insights into *in vivo* activities of lantibiotics from gallidermin and epidermin mode-of-action studies. Antimicrob Agents Chemother.

[51] Escano J, Stauffer B, Brennan J, Bullock M, Smith L (2014). The leader peptide of mutacin 1140 has distinct structural components compared to related class I lantibiotics. Microbiologyopen.

[52] Maffioli SI (2015). Family of class I lantibiotics from actinomycetes and improvement of their antibacterial activities. ACS Chem Biol.

[53] Mohr KI (2015). Pinensins: the first antifungal lantibiotics. Angew Chem Int Ed Engl.

[54] Garg N, Tang W, Goto Y, Nair SK, van der Donk WA (2012). Lantibiotics from Geobacillus thermodenitrificans. Proc Natl Acad Sci USA.

[55] Wescombe PA (2011). Salivaricin 9, a new lantibiotic produced by Streptococcus salivarius. Microbiology.

[56] Daly KM (2010). Production of the Bsa lantibiotic by community-acquired Staphylococcus aureus strains. J Bacteriol.

[57] Shi Y, Yang X, Garg N, van der Donk WA (2011). Production of lantipeptides in Escherichia coli. J Am Chem Soc.

[58] Eichenbaum Z (1998). Use of the *lactococcal* nisA promoter to regulate gene expression in gram-positive bacteria: comparison of induction level and promoter strength. Appl Environ Microbiol.

[59] Rollema HS, Kuipers OP, Both P, de Vos WM, Siezen RJ (1995). Improvement of solubility and stability of the antimicrobial peptide nisin by protein engineering. Appl Environ Microbiol.

[60] Healy B (2013). Intensive mutagenesis of the nisin hinge leads to the rational design of enhanced derivatives. PLoS One.

[61] Zhou L, van Heel AJ, Kuipers OP (2015). The length of a lantibiotic hinge region has profound influence on antimicrobial activity and host specificity. Front Microbiol.

[62] Breukink E (1998). The orientation of nisin in membranes. Biochemistry.

[63] Lubelski J, Khusainov R, Kuipers OP (2009). Directionality and coordination of dehydration and ring formation during biosynthesis of the lantibiotic nisin. J Biol Chem.

[64] Karakas Sen A (1999). Post-translational modification of nisin. The involvement of NisB in the dehydration process. Eur. J. Biochem..

[65] Wiegand I, Hilpert K, Hancock REW (2008). Agar and broth dilution methods to determine the minimal inhibitory concentration (MIC) of antimicrobial substances. Nature Protocols.

[66] Khosa S (2016). Structural basis of lantibiotic recognition by the nisin resistance protein from Streptococcus agalactiae. Sci Rep.

[67] Stein T, Heinzmann S, Solovieva I, Entian KD (2003). Function of Lactococcus lactis nisin immunity genes nisI and nisFEG after coordinated expression in the surrogate host Bacillus subtilis. J Biol Chem.

[68] Hacker C (2015). The Solution Structure of the Lantibiotic Immunity Protein NisI and Its Interactions with Nisin. J Biol Chem.

[69] Lagedroste M, Smits SHJ, Schmitt L (2017). Substrate Specificity of the Secreted Nisin Leader Peptidase NisP. Biochemistry.

[70] Schagger H (2006). Tricine-SDS-PAGE. Nat. Protoc..

[71] Khosa S, Lagedroste M, Smits SH (2016). Protein Defense Systems against the Lantibiotic Nisin: Function of the Immunity Protein NisI and the Resistance Protein NSR. Front Microbiol.

[72] Wu J, Watson JT (1998). Optimization of the cleavage reaction for cyanylated cysteinyl proteins for efficient and simplified mass mapping. Anal Biochem.

