# Supplementary material for: Systematic characterization of position one variants within the lantibiotic nisin
Source: Sci Rep. 2019 Jan 30;9:935. doi: 10.1038/s41598-018-37532-4 (PMC6353901; doi:10.1038/s41598-018-37532-4)
Supplement: Supplementary file 1 — Dataset 1 [file 41598_2018_37532_MOESM1_ESM.pdf]

Supplemental information

Systematic characterization of position one variants within the lantibiotic nisin

Marcel Lagedroste*, Jens Reiners*, Sander H.J. Smits#

and Lutz Schmitt #

Institute of Biochemistry, Heinrich-Heine-University Duesseldorf, Universitaetsstrasse 1,

40225, Duesseldorf, Germany.

*Contributed equally

#Address correspondence to Lutz Schmitt: lutz.schmitt@hhu.de and Sander H.J. Smits: sander.smits@hhu.de

Key words: lantibiotic, nisin, MS analysis, antimicrobial activity

Figures of supplemental information:

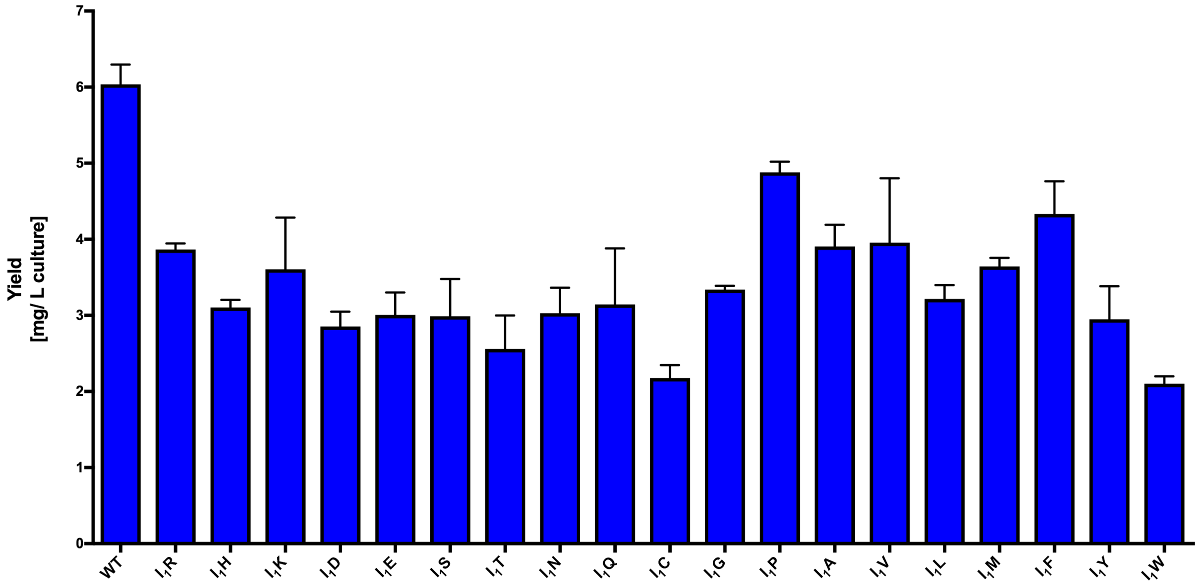

Figure S1: The yield per liter cell culture of pre-nisin variants

Summary of the yields per liter cell culture supernatant after cation-exchange chromatography of nisin A and their corresponding I_1 mutants. The purified peptides were quantified via RP-HPLC. Error bars represent the standard deviation of at least three biological replicates.

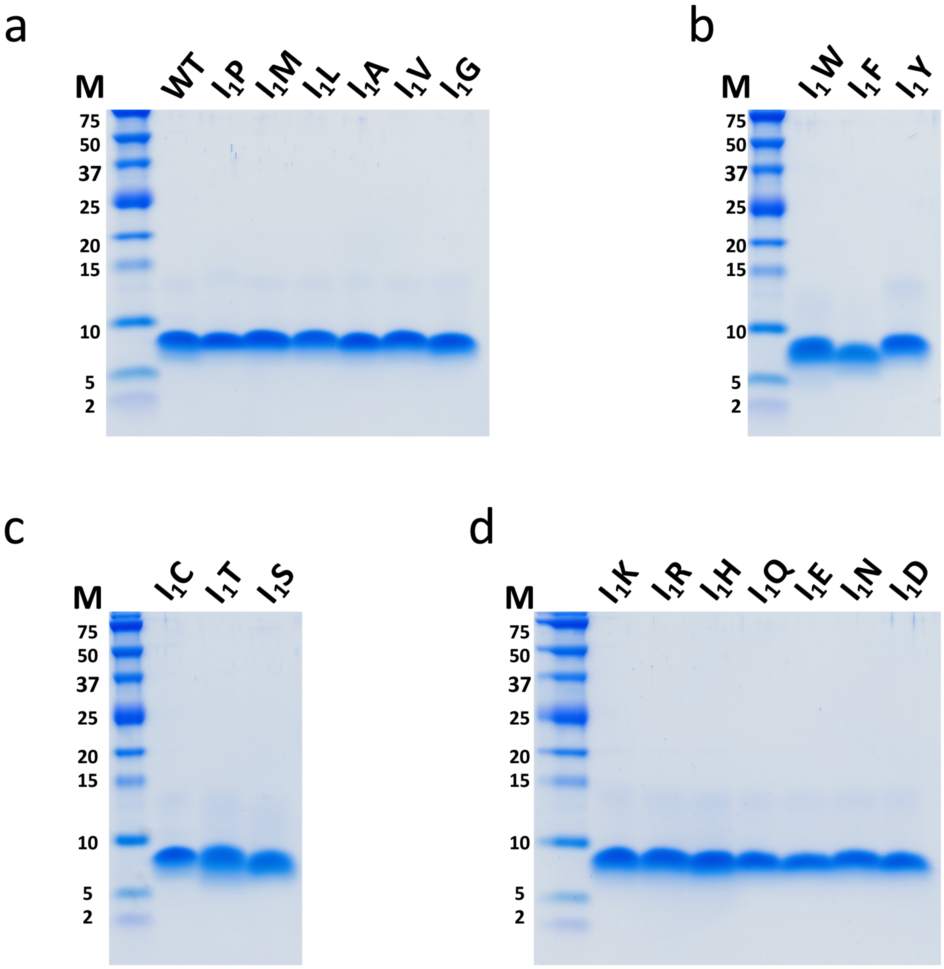

Figure S2: Tricine-SDS-Gels of pre-nisin, I_1 mutants.
Group 1 contained amino acids M-G (**a**), group 2 amino acids W-Y (**a**), group 3 with the amino acids C-S (**a**) and group 4 the amino acids K-H (**a**).

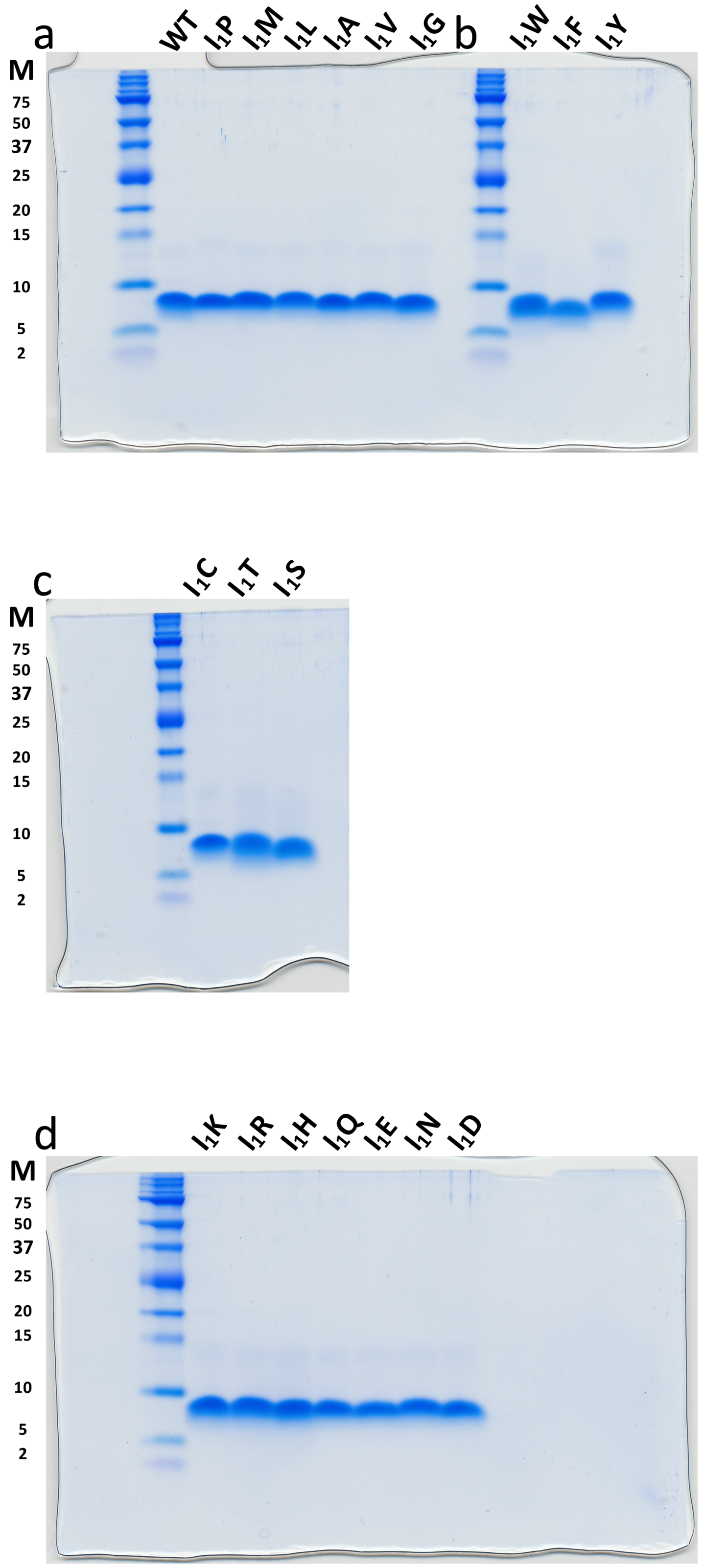

Figure S2: Original Tricine-SDS-Gels of pre-nisin, I_1 mutants.

Group 1 contained amino acids M-G (**a**), group 2 amino acids W-Y (**a**), group 3 with the amino acids C-S (**a**) and group 4 the amino acids K-H (**d**).

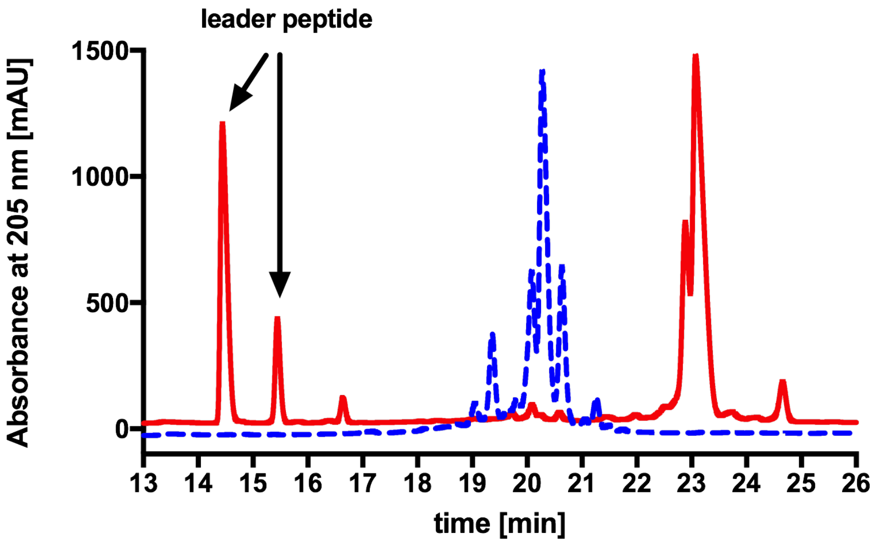

Figure S3: RP-HPLC chromatogram of pre-nisin and nisin.
The retention profile (min) of pre-nisin WT is shown in blue and the product of the cleavage reaction is shown in red. The black arrows indicate the leader peptide peaks (± N-terminal methionine) of cleaved pre-nisin, which is used for quantification.

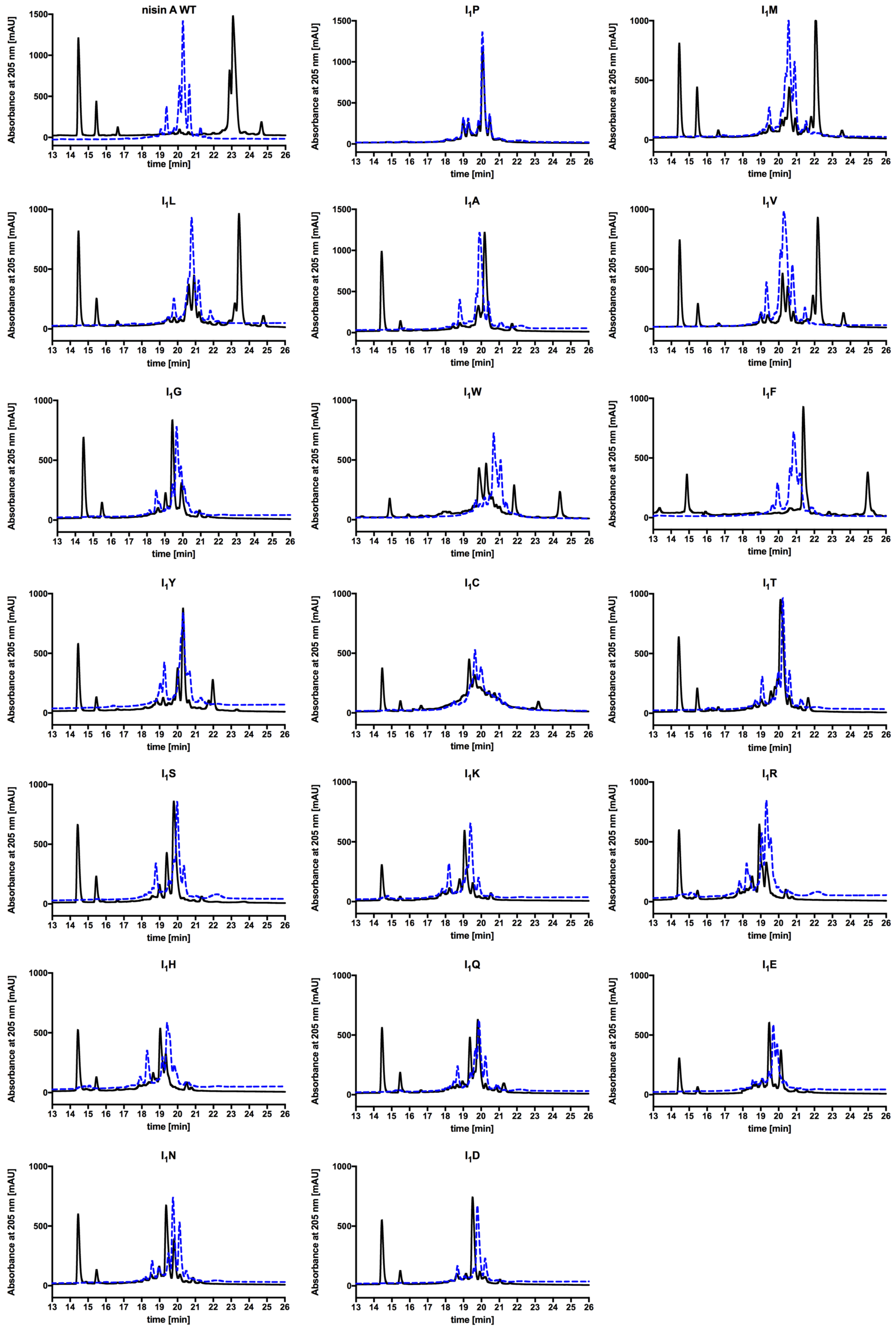

Figure S4: RP-HPLC chromatograms of the cleaved pre-nisin and I_1 mutants

The RP-HPLC chromatogram summaries all pre-nisin and I_1 mutants, which were used in this study. The pre-nisin variants were analysed before cleavage (blue dotted line) and after NisP cleavage (black line). Please note that the scaling of the y axis is different for nisin A WT, I_1P and I_1A.

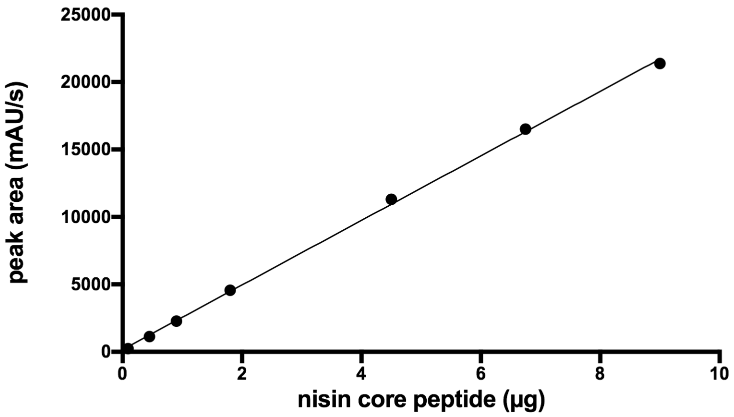

Figure S5: Calibration line of the nisin core peptide.
The slope of the calibration line makes it possible to quantify the total yield and the cleavage efficiency of NisP. Slope: 2392 ± 30.16, R^2: 0.9992

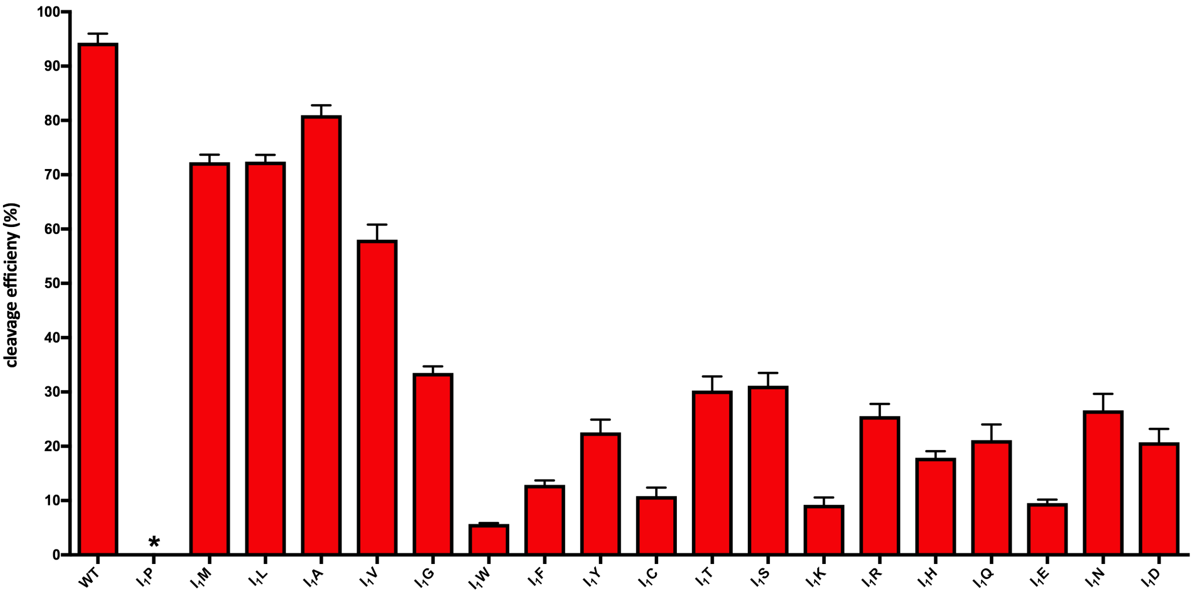

Figure S6: Quantification of the cleavage efficiency of NisP by RP-HPLC.
The leader peptide peak areas were used to determine the final concentration of the activated species and to calculate the efficiency (example see Supplementary Fig. S3, red line). The nisin mutant I_1P was not cleaved by NisP (*). Error bars represent the standard deviation of at least three biological replicates.

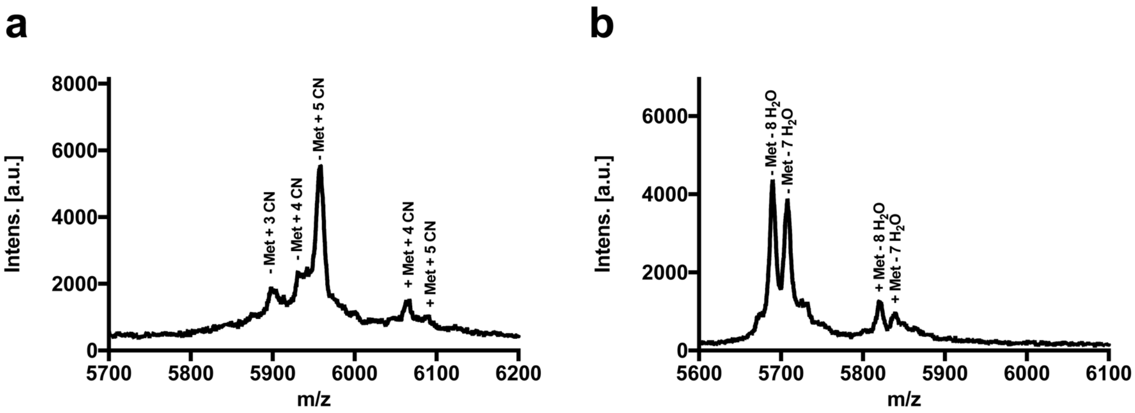

Figure S7: MALDI-TOF analysis to determine the level of dehydrations and ring formations

To demonstrate the specificity of the CDAP coupling, we threated unmodified pre-nisin and fully modified pre-nisin. **a**: unmodified pre-nisin with a maximum number of five coupling products, which demonstrated the accessibility of all five cysteine residues. The fully modified pre-nisin showed no coupling products indicating that all cysteine residues were involved in lanthionine rings (**b**).

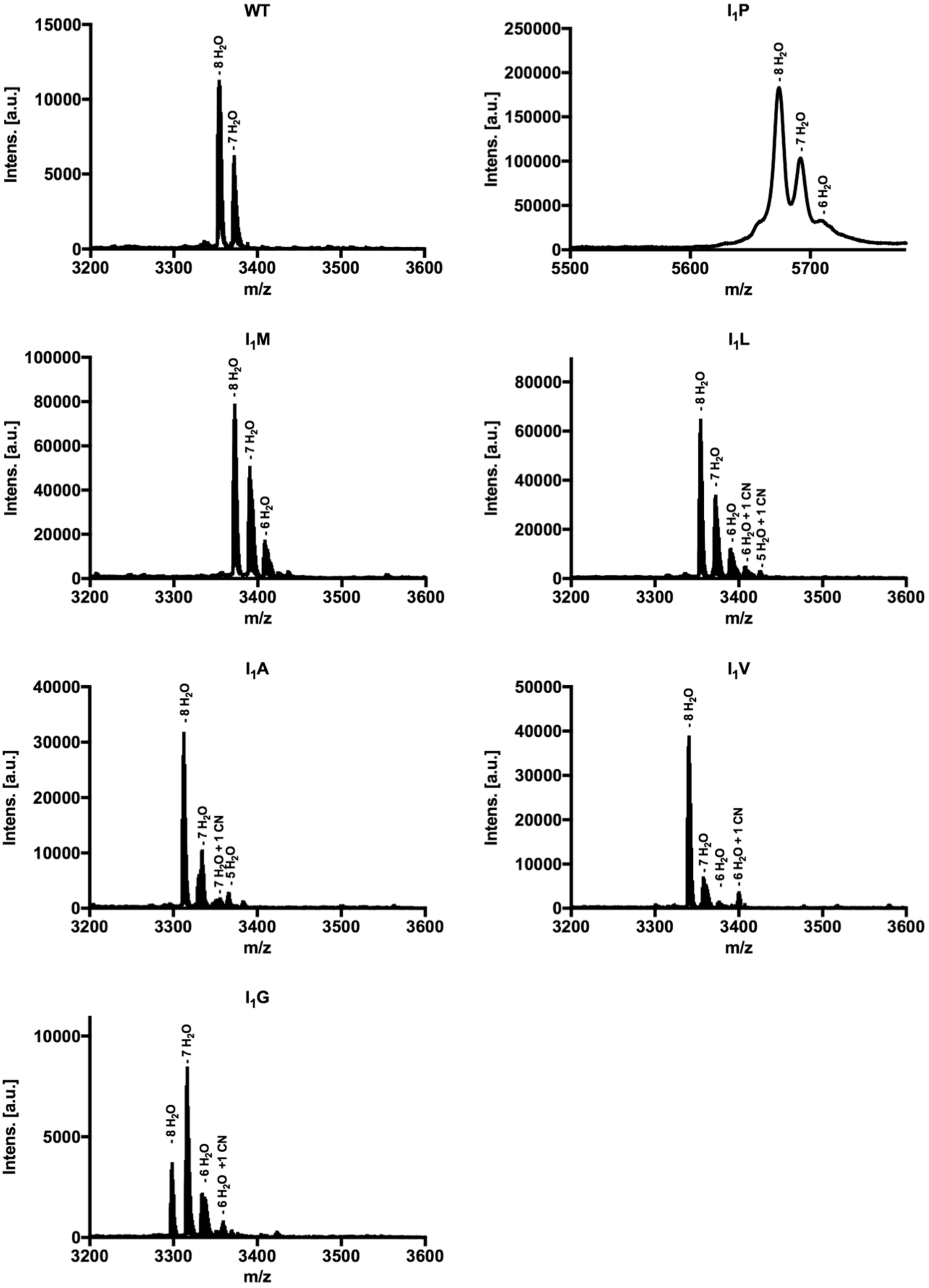

Figure S8: MALDI-TOF analysis to determine the level of dehydrations and ring formations for the nisin A variant group 1.

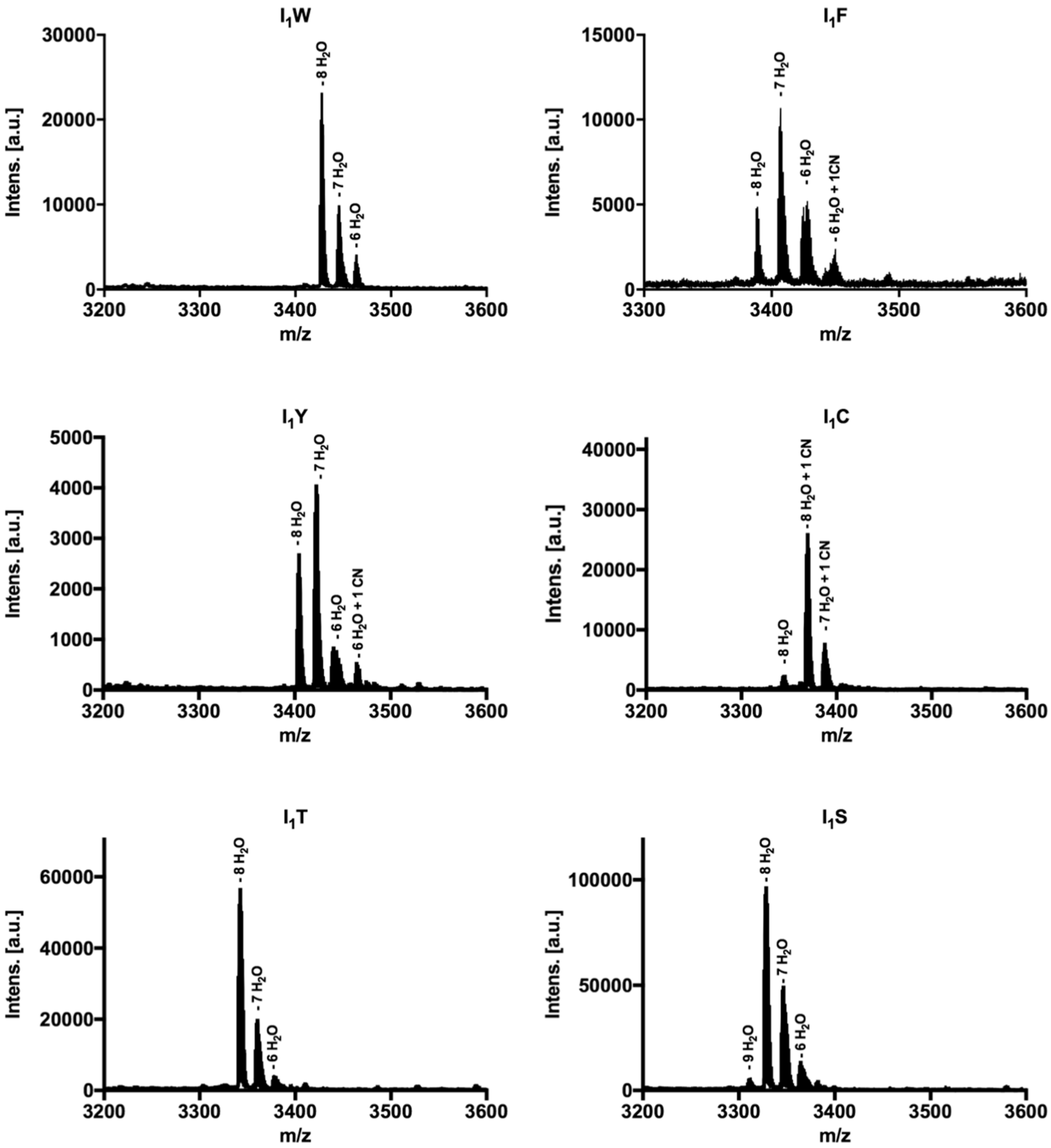

Figure S9: MALDI-TOF analysis to determine the level of dehydrations and ring formations for the nisin A variant group 2 and 3.

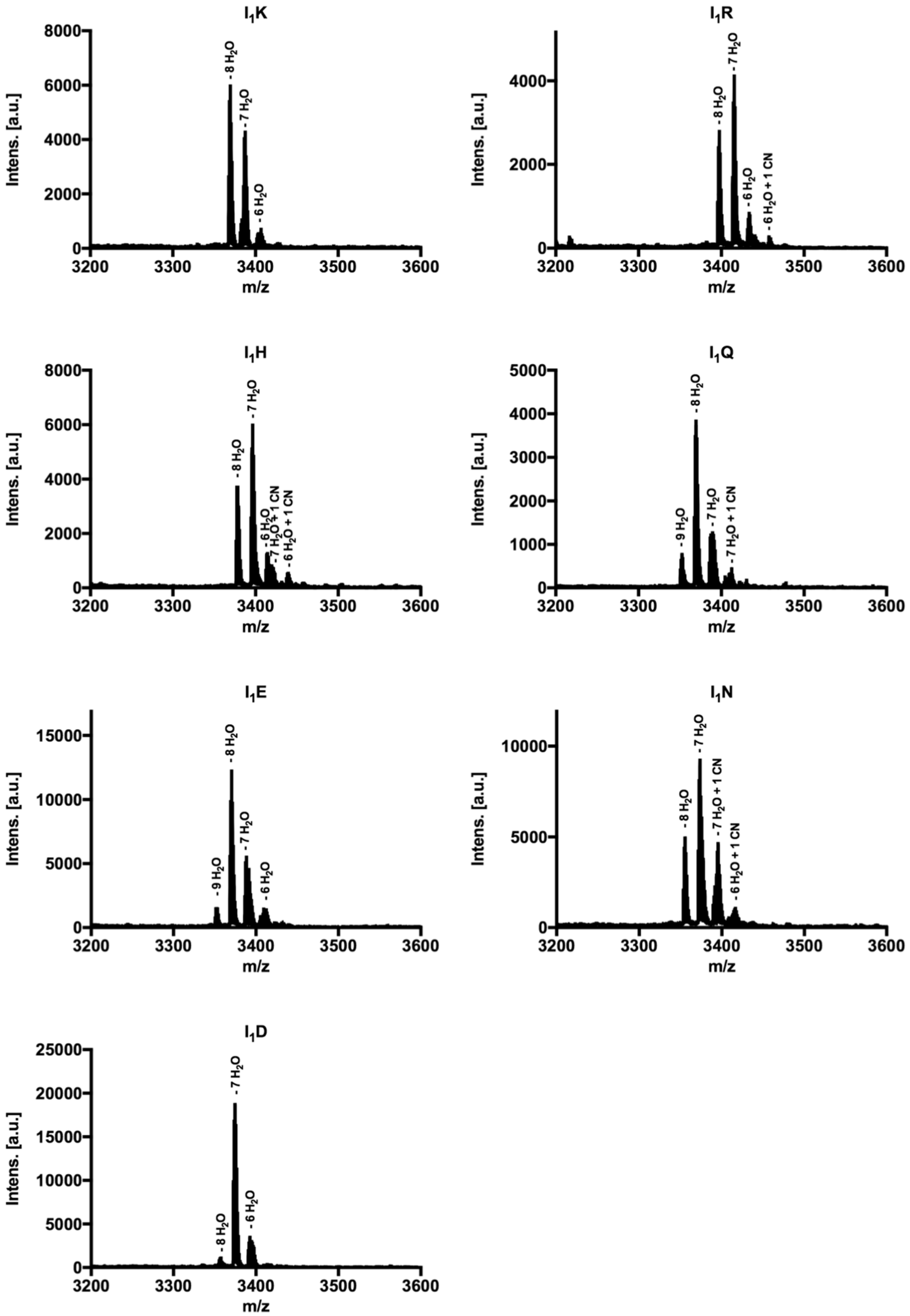

Figure S10: MALDI-TOF analysis to determine the level of dehydrations and ring formations for the nisin A variant group 4.

Tables of supplemental information:

Table S1: Overall data from the MS analysis of nisin A variants

	Variant	Observed masses [Da]	Modification
Group 1	WT	3354<br>3372	- 8 H_2O<br>- 7 H_2O
	I_1P	5672<br>5690<br>5708	Uncleaved + Met - 8 H_2O<br>Uncleaved + Met - 7 H_2O<br>Uncleaved + Met - 6 H_2O
	I_1M	3372<br>3390<br>3408	- 8 H_2O<br>- 7 H_2O<br>- 6 H_2O
	I_1L	3354<br>3372<br>3390<br>3415<br>3433	- 8 H_2O<br>- 7 H_2O<br>- 6 H_2O<br>- 6 H_2O + 1 CN<br>- 5 H_2O + 1 CN
	I_1A	3312<br>3330<br>3355<br>3366	- 8 H_2O<br>- 7 H_2O<br>- 7 H_2O + 1 CN<br>- 5 H_2O
	I_1V	3340<br>3358<br>3376<br>3401	- 8 H_2O<br>- 7 H_2O<br>- 6 H_2O<br>- 6 H_2O + 1 CN
	I_1G	3298<br>3316<br>3334<br>3359	- 8 H_2O<br>- 7 H_2O<br>- 6 H_2O<br>- 6 H_2O + 1 CN
Group 2	I_1W	3427<br>3445<br>3463	- 8 H_2O<br>- 7 H_2O<br>- 6 H_2O
	I_1F	3388<br>3406<br>3424<br>3449	- 8 H_2O<br>- 7 H_2O<br>- 6 H_2O<br>- 6 H_2O + 1 CN
	I_1Y	3404<br>3422<br>3440<br>3465	- 8 H_2O<br>- 7 H_2O<br>- 6 H_2O<br>- 6 H_2O + 1 CN
Group 3	I_1C	3344<br>3369<br>3387	- 8 H_2O<br>- 8 H_2O + 1 CN<br>- 7 H_2O + 1 CN
	I_1T	3342<br>3360<br>3378	- 8 H_2O<br>- 7 H_2O<br>- 6 H_2O
	I_1S	3310<br>3328<br>3346<br>3364	- 9 H_2O<br>- 8 H_2O<br>- 7 H_2O<br>- 6 H_2O
Group 4	I_1K	3369<br>3387<br>3405	- 8 H_2O<br>- 7 H_2O<br>- 6 H_2O
	I_1R	3397<br>3415<br>3433<br>3458	- 8 H_2O<br>- 7 H_2O<br>- 6 H_2O<br>- 6 H_2O + 1 CN
	I_1H	3378<br>3396	- 8 H_2O<br>- 7 H_2O

		**3414**<br>**3421**<br>**3439**	**- 6 H_2O**<br>**- 7 H_2O + 1 CN**<br>**- 6 H_2O + 1 CN**
	**I_1Q**	**3351**<br>**3369**<br>**3387**<br>**3412**	**- 9 H_2O**<br>**- 8 H_2O**<br>**- 7 H_2O**<br>**- 7 H_2O + 1 CN**
	**I_1E**	**3352**<br>**3370**<br>**3388**<br>**3406**	**- 9 H_2O**<br>**- 8 H_2O**<br>**- 7 H_2O**<br>**- 6 H_2O**
	**I_1N**	**3355**<br>**3373**<br>**3398**<br>**3416**	**- 8 H_2O**<br>**- 7 H_2O**<br>**- 7 H_2O + 1 CN**<br>**- 6 H_2O + 1 CN**
	**I_1D**	**3356**<br>**3374**<br>**3392**	**- 8 H_2O**<br>**- 7 H_2O**<br>**- 6 H_2O**

Table S2: IC_{50} values of nisin A and variants

IC_{50} values were determined against the sensitive strain NZ9000-Cm and against strain NZ9000 expressing the immunity/resistance proteins NisI, NisFEG, *Sa*NSR and *Sa*NsrFP.

	**NZ9000-Cm**	**NZ9000-NisI**	**NZ9000-NisFEG**	**NZ9000-*Sa*NSR**	**NZ9000-*Sa*NsrFP**
	IC_{50} [nM]	IC_{50} [nM]	IC_{50} [nM]	IC_{50} [nM]	IC_{50} [nM]
**WT**	4.8 ± 0.7	46.0 ± 6.0	53.0 ± 4.5	73.1 ± 3.6	82.1 ± 3.7
**I_1P**	not cleavable				
**I_1M**	5.8 ± 0.3	51.5 ± 1.6	39.2 ± 1.0	84.5 ± 1.7	64.9 ± 0.7
**I_1L**	9.8 ± 0.5	35.5 ± 1.5	34.6 ± 1.2	53.0 ± 0.4	50.4 ± 0.6
**I_1A**	10.7 ± 0.4	65.0 ± 2.8	34.2 ± 1.0	68.0 ± 1.9	166.1 ± 5.1
**I_1V**	11.8 ± 0.9	43.8 ± 1.7	50.4 ± 0.3	66.8 ± 0.34	59.2 ± 2.2
**I_1G**	143.0 ± 5.1	785.7 ± 9.7	557.8 ± 28.3	278.5 ± 13.3	2257.0 ± 53.4
**I_1W**	2.5 ± 0.2	22.7 ± 1.5	21.7 ± 1.2	18.3 ± 0.7	46.6 ± 1.1
**I_1F**	3.7 ± 0.8	36.5 ± 3.3	25.9 ± 1.9	43.9 ± 1.2	64.3 ± 0.4
**I_1Y**	10.6 ± 0.9	84.3 ± 1.1	45.7 ± 1.8	105.7 ± 1.3	137.9 ± 4.1
**I_1C**	8.6 ± 0.5	55.1 ± 2.3	41.7 ± 0.7	101.4 ± 3.8	68.8 ± 3.2
**I_1T**	37.3 ± 1.6	653.3 ± 5.1	107.7 ± 6.2	130.8 ± 3.4	716.1 ± 28.6
**I_1S**	112.4 ± 5.0	1898.0 ± 62.3	337.3 ± 17.5	253.1 ± 6.2	2893.0 ± 34.8
**I_1K**	44.7 ± 3.0	435.7 ± 9.9	301.2 ± 4.7	171.7 ± 0.8	983.6 ± 16.2
**I_1R**	113.9 ± 13.6	422.9 ± 20.4	186.4 ± 3.1	227.0 ± 5.6	1153.0 ± 31.0
**I_1H**	140.0 ± 5.0	1488.0 ± 85.1	1172.0 ± 134.3	232.9 ± 3.4	3213.0 ± 19.1
**I_1Q**	592.0 ± 17.8	n.d.			
**I_1E**	1328.0 ± 32.7				
**I_1N**	1386.0 ± 46.3				
**I_1D**	3746.0 ± 144.1				

n.d.: not determined.

Table S3: Nomenclature of the strain NZ9000 expressing immunity (NisI and NisFEG) and resistance proteins (*Sa*NSR and *Sa*NsrFP)

Strain name	plasmid	Expressed protein	properties	Ref.
NZ9000	-	-	sensitive *Lactococcus lactis* strain lacking the gens for *NisABTCPIFEG*	1
NZ9000-Erm	pNZ-SV Erm	-	erythromycin resistance; sensitiv strain	2
NZ9000-Cm	pIL-SV Cm	-	chloramphenicol resistance; sensitiv strain	3
NZ9000-NisP	pNG-nisP8His	NisP	chloramphenicol resistance; sensitive strain expressing the peptidase NisP from *Lactococcus lactis*	4
NZ9000-NisBTC	pIL3-BTC	NisBTC	chloramphenicol resistance; sensitive strain expressing the modification proteins NisB, NisC and the ABC transporter NisT from *Lactococcus lactis*	5
NZ9000-NisI	pNZ-SV-nisI	NisI	erythromycin resistance; immunity strain expressing the lipoprotein NisI from *Lactococcus lactis*	2
NZ9000-NisFEG	pIL-SV-nisFEG	NisFEG	chloramphenicol resistance; immunity strain expressing the ABC transporter NisFEG from *Lactococcus lactis*	3
NZ9000-*Sa*NSR	pNZ-SV-nsr	*Sa*NSR	erythromycin resistance; resistance strain expressing the nisin peptidase NSR from *Streptococcus agalactiae*	6
NZ9000-*Sa*NsrFP	pIL-SV-nsrFP	*Sa*NsrFP	chloramphenicol resistance; resistance strain expressing the BceAB-type ABC transporter NsrFP from *Streptococcus agalactiae*	7

Table S4: Primer sequences used for site-directed mutagenesis

The primer pairs were used for the point mutations at position 1 in the core peptide of nisin A. The exchanged codon is labelled red within the forward (fw) and reversed (rw) primer sequences.

Oligonucleotide name	Sequence (5'-3')
I-K fw	GTGCATCACCACGCAAAACAAGTATTTCGC
I-K rw	GCGAAATACTTGTTTTGCGTGGTGATGCAC
I-M fw	GTGCATCACCACGCATGACAAGTATTTCGC
I-M rw	GCGAAATACTTGTCATGCGTGGTGATGCAC
I-N fw	GTGCATCACCACGCAATACAAGTATTTCGC
I-N rw	GCGAAATACTTGTATTGCGTGGTGATGCAC
I-R fw	GTGCATCACCACGCAGAACAAGTATTTCGC
I-R rw	GCGAAATACTTGTTCTGCGTGGTGATGCAC
I-S fw	GTGCATCACCACGCAGTACAAGTATTTCGC
I-S rw	GCGAAATACTTGTACTGCGTGGTGATGCAC
I-T fw	GTGCATCACCACGCACAACAAGTATTTCGC
I-T rw	GCGAAATACTTGTTGTGCGTGGTGATGCAC
I-V fw	GTGCATCACCACGCGTTACAAGTATTTCGC
I-V rw	GCGAAATACTTGTAACGCGTGGTGATGCAC
I-A fw	GTGCATCACCACGCGCTACAAGTATTTCGC
I-A rw	GCGAAATACTTGTAGCGCGTGGTGATGCAC
I-D fw	GTGCATCACCACGCGATACAAGTATTTCGC
I-D rw	GCGAAATACTTGTATCGCGTGGTGATGCAC
I-E fw	GTGCATCACCACGCGAAACAAGTATTTCGC
I-E rw	GCGAAATACTTGTTTCGCGTGGTGATGCAC
I-G fw	GTGCATCACCACGCGGTACAAGTATTTCGC
I-G rw	GCGAAATACTTGTACCGCGTGGTGATGCAC
I-L fw	GTGCATCACCACGCTTAACAAGTATTTCGC
I-L rw	GCGAAATACTTGTTAAGCGTGGTGATGCAC
I-Y fw	GTGCATCACCACGCTATACAAGTATTTCGC
I-Y rw	GCGAAATACTTGTATAGCGTGGTGATGCAC

I-C fw	GTGCATCACCACGCTGTACAAGTATTTCGC
I-C rw	GCGAAATACTTGTACAGCGTGGTGATGCAC
I-W fw	GTGCATCACCACGCTGGACAAGTATTTCGC
I-W rw	GCGAAATACTTGTCCAGCGTGGTGATGCAC
I-P fw	GTGCATCACCACGCCCAACAAGTATTTCGC
I-P rw	GCGAAATACTTGTTGGGCGTGGTGATGCAC
I-H fw	GTGCATCACCACGCCATACAAGTATTTCGC
I-H rw	GCGAAATACTTGTATGGCGTGGTGATGCAC
I-Q fw	GTGCATCACCACGCCAAACAAGTATTTCGC
I-Q rw	GCGAAATACTTGTTTGGCGTGGTGATGCAC
I-F fw	CATCACCACGCTTTACAAGTATTTCGC
I-F rw	GCGAAATACTTGTAAAGCGTGGTGATG

References:

1. de Ruyter, P.G., Kuipers, O.P. & de Vos, W.M. Controlled gene expression systems for Lactococcus lactis with the food-grade inducer nisin. *Appl Environ Microbiol* **62**, 3662-7 (1996).
2. AlKhatib, Z. et al. Lantibiotic immunity: inhibition of nisin mediated pore formation by NisI. *PLoS One* **9**, e102246 (2014).
3. AlKhatib, Z. et al. The C-terminus of nisin is important for the ABC transporter NisFEG to confer immunity in Lactococcus lactis. *Microbiologyopen* **3**, 752-63 (2014).
4. Abts, A., Montalban-Lopez, M., Kuipers, O.P., Smits, S.H. & Schmitt, L. NisC binds the FxLx motif of the nisin leader peptide. *Biochemistry* **52**, 5387-95 (2013).
5. Rink, R. et al. Lantibiotic structures as guidelines for the design of peptides that can be modified by lantibiotic enzymes. *Biochemistry* **44**, 8873-82 (2005).
6. Khosa, S. et al. Structural basis of lantibiotic recognition by the nisin resistance protein from Streptococcus agalactiae. *Sci Rep* **6**, 18679 (2016).
7. Reiners, J., Abts, A., Clemens, R., Smits, S.H. & Schmitt, L. Stoichiometry and structure of a lantibiotic maturation complex. *Sci Rep* **7**, 42163 (2017).
